# Persistence and stability of generalized ribosome flow models with time-varying transition rates

**DOI:** 10.1371/journal.pone.0288148

**Published:** 2023-07-07

**Authors:** Mihály A. Vághy, Gábor Szederkényi

**Affiliations:** 1 Faculty of Information Technology and Bionics, Pázmány Péter Catholic University, Budapest, Hungary; 2 Systems and Control Laboratory, Institute for Computer Science and Control (SZTAKI), Budapest, Hungary; Institute of Space Technology, PAKISTAN

## Abstract

In this paper some important qualitative dynamical properties of generalized ribosome flow models are studied. Ribosome flow models known from the literature are generalized by allowing an arbitrary directed network structure between compartments, and by assuming general time-varying rate functions corresponding to the transitions. Persistence of the dynamics is shown using the chemical reaction network (CRN) representation of the system where the state variables correspond to ribosome density and the amount of free space in the compartments. The L1 contractivity of solutions is also proved in the case of periodic reaction rates having the same period. Further we prove the stability of different compartmental structures including strongly connected ones with entropy-like logarithmic Lyapunov functions through embedding the model into a weakly reversible CRN with time-varying reaction rates in a reduced state space. Moreover, it is shown that different Lyapunov functions may be assigned to the same model depending on the non-unique factorization of the reaction rates. The results are illustrated through several examples with biological meaning including the classical ribosome flow model on a ring.

## 1 Introduction

Compartmental models are used to describe and analyze the transport between different containers, called compartments in various natural and technological systems [1, 2]. Compartments can be assigned to tissues or organs in pharmacokinetic models, mass containers in process systems, distinct disease states in epidemiological models, road sections in transportation systems or different habitats in ecological models. The modeled objects (molecules, people, vehicles, etc.) can move between compartments obeying the given constraints such as limits of directions, flow rates, or capacities. A fundamental feature of compartmental models is that each modeled object can be present in exactly one compartment at a given time. Naturally, compartmental models written in the original physical coordinates belong to the class of nonnegative systems for which the nonnegative orthant is invariant with respect to the dynamics [3, 4]. This special property supports the dynamical analysis and control design in several ways. The controllability, observability, realizability and identifiability of mainly linear compartmental system are addressed in [5]. An excellent overview of the qualitative dynamical properties of general compartmental systems can be found in [6].

The dynamical modeling of the mRNA translation process has been in the focus of research since the second half of the 20th century (see, e.g. [7–9]). The first large scale analysis of gene translation through the so-called ribosome flow model (RFM) was presented in [10], where the applied second order nonnegative and nonlinear model based on the principle of Totally Asymmetric Exclusion [11] was able to capture the most important dynamical features of the translation process. Also in [10], the RFM model was validated through biological data obtained from three different organisms, and it was clearly shown that its predictive power is superior to several other popular techniques. In [12] the RFM was equipped with an appropriate input-output pair, and it was shown that after applying an affine positive output feedback, the system had a unique equilibrium point which is globally stable in the bounded operating domain. A circular RFM structure was analyzed in [13], where the authors proved using the theory of cooperative systems that the system has a continuum of equilibria, but each equilibrium is globally asymptotically stable within the equivalence class of trajectories determined by the initial conditions. The stability of periodic solutions was also shown. In [14] a bounded pool of free ribosomes was added to the RFM generating a competition among the arbitrary number of mRNA molecules for ribosomes. This generates a special network structure for RFM subsystems, for which the uniqueness and stability of equilibria together with the properties of periodic solutions were proved, too. Different compartment sizes of the RFM were assumed in [15], and it was shown that this modification does not change the favorable dynamical properties of the system. In [16], the ribosome flow model with Langmuir kinetics (RFMLK) is introduced, and a network structure is constructed with RFMLK subsystems connected through a pool. Among other results, it is shown that the trajectories of such a network always converge to a unique equilibrium.

Chemical reaction networks (CRNs) also called kinetic systems can be considered as universal descriptors of nonlinear dynamics, especially that of nonnegative systems [17]. Since the 1970’s the theory of CRNs has been intensively studied, and there are several fundamental results on the relation between network structure/parametrization and dynamical properties [18]. The stability of mass-action type CRNs is most often analyzed using an entropy-like logarithmic Lyapunov function, originally called a “pseudo-Helmholtz function” in [19]. Probably the most well-known conjecture of chemical reaction network theory is the “Global attractor conjecture” according to which complex balanced kinetic systems are globally stable with respect to the nonnegative orthant with the logarithmic Lyapunov function [20]. This conjecture was proved for complex balanced reaction networks with a reaction graph of one component [21]. One of the most important results from the point of view of this paper is [22] studying zero deficiency networks, where the allowed kinetics is more general than mass action, the rate coefficients can be time-varying, and the logarithmic Lyapunov function is also generalized. The Lyapunov-function-based stability analysis of RFMs is mentioned as an important problem in [23], which will be addressed in this paper using the CRN representation of the system. In [24] a so-called Max-Min type robust Lyapunov function composed of piecewise linear terms was constructed for a tubular RFM with mass action kinetics.

It is interesting to mention that mathematical models which are equivalent to RFMs can also be obtained through a special finite volume spatial discretization of widely used flow models in PDE form [25, 26]. These models also have a transparent representation in CRN form supporting further dynamical analysis. An arbitrary directed graph structure of such models with general time-invariant kinetics was considered in [27], where the existence and uniqueness of equlibria, persistence and contractivity (non-expansive property) of the solutions was shown using the theory of Petri nets, compartmental systems, and earlier results on RFMs. The stability of this model class with logarithmic Lyapunov functions was shown in [28], while a port-Hamiltonian description was given in [29].

Based on the above overview, the aim of this paper is to extend the results of [27, 28] in the following respects: considering even more general kinetics with explicit time-dependence, the qualitative analysis of periodic solutions, and finally, stability analysis with a family of different logarithmic Lyapunov functions.

The structure of the paper is the following. Section 2 contains the applied mathematical notations for compartmental models and kinetic systems. In Section 3 the kinetic representation of the studied model class is described, while new results on persistence and periodic behaviour in the time-varying case are proposed in Section 4. Stability analysis results with a family of non-unique logarithmic Lyapunov functions are described in Section 5, and finally, Section 6 summarizes the main results of the paper.

## 2 Notations and background

In this section, we describe the basic notations and building blocks of a compartmental system class and chemical reaction networks (CRNs). The notations and overview in this section are based on [27, 29].

### 2.1 Compartmental models

Throughout the paper we consider systems containing a set of interconnected compartments and objects (such as ribosomes, particles, molecules, vehicles etc.) moving between them. We assume that the rate of transfer between compartments depends on the amount of objects in the source compartment as well as on the amount of free space in the target compartment. This naturally implies that each compartment has a well-defined finite *capacity* that limits the amount of modeled quantities that can be contained in the given compartment. We also allow explicit time dependence and in some cases dependence on the amount of objects and free space in other compartments.

For the formal definition, let us consider the set *Q* = {*q*_1_, *q*_2_, …, *q*_*m*_} of compartments and the set *A* ⊂ *Q* × *Q* of transitions, where (*q*_*i*_, *q*_*j*_) ∈ *A* represents the transition from compartment *q*_*i*_ into *q*_*j*_. Then, the directed graph *D* = (*Q*, *A*) is called the *compartmental graph* and it describes the structure of the compartmental model. The transitions are assumed to be immediate, thus loop edges are not allowed in the model since they do not introduce additional dynamical terms. Similarly, we do not allow parallel edges between two compartments in the same direction since they can be replaced by a single transition. We say that a (compartmental) graph is *strongly connected* if there exists a directed path between any two vertices in both directions, and we say that a graph is *weakly reversible* if it is a collection of isolated strongly connected subgraphs.

For each compartment *q*_*i*_ we introduce the sets of *donors* and *receptors*, respectively, as

$$\begin{array}{c} \begin{array}{cc} D_{i} & =\left\{j\in \left\{1,2,\cdots ,m\right\}|(q_{j},q_{i})\in A\right\},\\ R_{i} & =\left\{j\in \left\{1,2,\cdots ,m\right\}|(q_{i},q_{j})\in A\right\}; \end{array} \end{array}$$ (1)

that is, the set of donors of a given compartment are the compartments where an incoming transition originates from and the set of receptors are the compartments where an outgoing transition terminates in.

### 2.2 Chemical reaction networks (kinetic systems)

In this subsection we give a brief introduction of kinetic systems based on [18, 19], where more details can be found. A chemical reaction network (CRN) contains a set of *species* Σ = {*X*_1_, *X*_2_, …, *X*_*N*_} and the corresponding species vector is given by *X* = [*X*_1_ *X*_2_ … *X*_*N*_]^T^. The species of a CRN are transformed into each other through *elementary reaction steps* of the form

$$\begin{array}{c} C_{j}\overset{K_{j}\left(t\right)}{\to }C_{j^{\prime }}\;j=1,2,\cdots ,R, \end{array}$$ (2)

where $C_{j}=y_{j}^{T}X$ and $C_{j^{\prime }}=y_{j^{\prime }}^{T}X$ are the source and product *complexes*, respectively, the vectors $y_{j},y_{j^{\prime }}\in N_{0}^{N}$ are *stoichiometric coefficient vectors* and functions $K_{j}:{\bar{R}}_{+}^{N}\times {\bar{R}}_{+}\mapsto {\bar{R}}_{+}$ are the *rate functions* with ${\bar{R}}_{+}$ denoting the set of nonnegative real numbers. The matrix *Y* containing the stoichiometric coefficient vectors as columns is called the *stoichiometric matrix*. The subspace $S\subset R^{N}$ spanned by the so-called *reaction vectors* *y*_*j*′_ − *y*_*j*_ is called the *stoichiometric subspace* of the CRN.

The CRN structure can be uniquely described by a directed graph as follows. For each complex we assign a vertex in the graph and for each elementary reaction step of the form *C*_*j*_ → *C*_*j*′_ we assign a directed edge between the corresponding vertices. We call the resulting graph the *reaction graph* of the CRN. The *deficiency* of the CRN is defined as *δ* = *m* − *ℓ* − *s*, where *m* is the number of distinct complexes, *ℓ* is the number of linkage classes (graph components) in the reaction graph and *s* is the dimension of the stoichiometric subspace.

Let $x\left(t\right)\in {\bar{R}}_{+}^{N}$ denote the state vector of the species as a function of time for *t* ≥ 0. Based on the above, the dynamics of the CRN is given by

$$\begin{array}{c} \dot{x}\left(t\right)=\sum_{j=1}^{R}K_{j}\left(x,t\right)\left[y_{j^{\prime }}-y_{j}\right]. \end{array}$$ (3)

We assume that a reaction can only take place if each species of the given reaction have nonzero concentration; that is, we assume that $K_{j}(x\left(t\right),t)=0$ whenever there exists *k* ∈ *supp*(*y*_*j*_) such that *x*_*k*_(*t*) = 0, where we say that *k* ∈ *supp*(*y*_*j*_) if [*y*_*j*_]_*k*_ > 0. This property ensures the invariance of the nonnegative orthant (or a part of it). We also presume standard regularity assumptions of the rate functions that guarantee local existence and uniqueness of solutions. Different results in this paper require different sets of such assumptions, thus for the sake of generality they will be specified later. Dynamics of the form of (3) is called *persistent* if no trajectory that starts in the positive orthant has an omega-limit point on the boundary of $R_{+}^{N}$.

We note that for any $v\in S^{⊥}$ (where S denotes the stoichiometric subspace) we have that

$$\begin{array}{c} \left\langle \dot{x},v\right\rangle =\sum_{j=1}^{R}K_{j}\left(x,t\right)\left\langle y_{j^{\prime }}-y_{j},v\right\rangle =0 \end{array}$$ (4)

and thus 〈*x*, *v*〉 is constant. Since $v\in S^{⊥}$ was arbitrary we have that x(t)∈x(0)+S. This shows that the translates of S define invariant linear manifolds for the system. We further define for each $p\in R_{+}^{n}$ a positive stoichiometric compatibility class $S_{p}=\left(p+S\right)\cap {\bar{R}}_{+}^{n}$.

A set of ODEs of the form $\dot{x}=f\left(x,t\right)$ is called kinetic if it can be written in the form (3) with appropriate rate functions and stoichiometric coefficient vectors.

## 3 Kinetic representation

In this section we construct a kinetic representation of the above compartmental system class. To do so, we assign a CRN that incorporates the compartmental structure. This allows the introduction of a system of ODEs of the form (3) describing the time evolution of the compartmental model. Some of the following steps are described in [27] or [29] in a time-invariant setting but here we recall and extend them for convenience.

### 3.1 Kinetic modeling of compartmental transitions

Let us consider a compartmental model *D* = (*Q*, *A*). Let the set of species be Σ = {*N*_1_, *N*_2_, …, *N*_*m*_} ∪ {*S*_1_, *S*_2_, …, *S*_*m*_} where *N*_*i*_ and *S*_*i*_ represent the number of particles and available spaces in compartment *q*_*i*_, respectively. To each transition (*q*_*i*_, *q*_*j*_) ∈ *A* we assign a reaction of the form (see, also [24])

$$\begin{array}{c} N_{i}+S_{j}\overset{K_{ij}}{\to }N_{j}+S_{i}, \end{array}$$ (5)

where $K_{ij}$ is the rate function of the transition. Such a reaction represents that during the transition from compartment *q*_*i*_ to compartment *q*_*j*_ the number of items decreases in *q*_*i*_ and increases in *q*_*j*_, while the number of available spaces increases in *q*_*i*_ and decreases in *q*_*j*_. Let *n*_*i*_ and *s*_*i*_ denote the continuous amount of particles and free space in *q*_*i*_, respectively.

Based on (3) the dynamics of the system is given by

$$\begin{array}{c} \begin{array}{cc} {\dot{n}}_{i} & =\sum_{j\in D_{i}}K_{ji}\left(n,s,t\right)-\sum_{j\in R_{i}}K_{ij}\left(n,s,t\right),\\ {\dot{s}}_{i} & =-\sum_{j\in D_{i}}K_{ji}\left(n,s,t\right)+\sum_{j\in R_{i}}K_{ij}\left(n,s,t\right) \end{array} \end{array}$$ (6)

where *n* and *s* denote the vectorized form of the variables *n*_*i*_ and *s*_*i*_, respectively. It is easy to check that the model class in Eq (6) contains ribosome flow models described in [23] or [15], and extends them in two ways: firstly, the reaction rate function K is not necessarily mass-action type and moreover, is time-varying, and secondly, the compartmental graph of the system can be arbitrary (i.e., there can be transitions between any two compartments). Note, that we also allow the transition rates to depend on the amount of objects and free space in other compartments as well, possibly describing inhibitory phenomena. Therefore, we call (6) a *generalized time-varying ribosome flow model*. Thus, our novel results not only extend the theory of ribosome flow models, but can be applied to other TASEP based transport models [30–34] and other flow models, such as the Traffic Reaction Model of [25] or the Nonlocal Flow Reaction Model of [26]. Finally, we note, that while more complicated network structures may not be biologically relevant in the case of ribosome flows, but can serve as a great tool for the analysis of other flow based physical models, e.g. traffic flows.

Clearly the reaction graph of the assigned CRN of a compartmental model is generally not strongly connected nor weakly reversible even if the compartmental graph is strongly connected. In fact, the reaction graph is weakly reversible if and only if each transition in the compartmental system is reversible. Even though the reaction graph, in some sense, loses the regularities of the compartmental graph, we can explicitly determine its deficiency from the compartmental topology and, as described in [27], CRNs of the form (6) exhibit persistence and stability properties in various senses in the time-invariant case.

### 3.2 Deficiency of CRNs realizing compartmental models

For a compartmental system *D* = (*Q*, *A*) let $\left|D\right|=(Q,\tilde{A})$ denote the undirected graph where the parallel edges are merged.

**Theorem 3.1** *The deficiency of a CRN assigned to a compartmental model D* = (*Q*, *A*) *is equal to the number of chordless cycles in the undirected graph* $\left|D\right|=(Q,\tilde{A})$.

*Proof*. For each transition between *q*_*i*_ and *q*_*j*_ we assign two complexes, namely *N*_*i*_ + *S*_*j*_ and *S*_*i*_ + *N*_*j*_, regardless of the transitions’ direction, so reversible reactions do not introduce additional complexes, and thus the number of stoichiometrically distinct complexes is $m=2|\tilde{A}|$. A complex of the form *N*_*i*_ + *S*_*j*_ is only connected with the complex *S*_*i*_ + *N*_*j*_, and thus we have $\ell =|\tilde{A}|$ linkage classes each consisting of exactly two complexes. To find the dimension of the stoichiometric subspace, denoted by s=dimS, observe that the reaction vector of a reaction of the form *N*_*i*_ + *S*_*j*_ → *N*_*j*_ + *S*_*i*_ is

$$\begin{array}{c} y_{i\to j}=-e_{i}+e_{j}+e_{m+i}-e_{m_{j}}, \end{array}$$ (7)

where $e_{k}\in R^{2m}$ denotes the *k*th unit vector. Again, since *y*_*i*→*j*_ = −*y*_*j*→*i*_ it suffices to consider the undirected graph |*D*|. Assume that *y*_*i*→*j*_ is such that

$$\begin{array}{c} y_{i\to j}=\sum c_{l\to l^{\prime }}y_{l\to l^{\prime }}. \end{array}$$ (8)

Then by (7) we have that for each non-zero term of the form *c*_.→*l*′_*y*_.→*l*′_ the right-hand side also contains at least one non-zero term *c*_*l*′→._*y*_*l*′→._, including the terms *c*_*i*→._*y*_*i*→._ and *c*_.→*j*_*y*_.→*j*_. This shows that the edges corresponding to the reaction vectors of the right-hand side form possibly multiple cycles in |*D*|. Without the loss of generality we may assume that this subgraph does not contain cycles isolated from (*q*_*i*_, *q*_*j*_). We have to consider the following cases:

1. First, we assume that the right-hand side is a single chordless cycle and contains the transitions
  $$\begin{array}{c} q_{i}\to q_{l_{1}}\to q_{l_{2}}\to \cdots \to q_{l_{r}}\to q_{j}\to q_{i}. \end{array}$$ (9)
  Taking the inner product of unit vectors $e_{i},e_{l_{1}},e_{l_{2}},\ldots ,e_{l_{r}},e_{j}$ and
  $$\begin{array}{c} y_{i\to j}=c_{i\to l_{1}}y_{i\to l_{1}}+\sum_{k=1}^{r-1}c_{l_{k}\to l_{k+1}}y_{l_{k}\to l_{k+1}}+c_{l_{r}\to j}y_{l_{r}\to j} \end{array}$$ (10)
  yields the system of linear equations:
  $$\begin{array}{c} \begin{array}{cc} -1 & =-c_{i\to l_{1}}\\ 0 & =c_{i\to l_{1}}-c_{l_{1}\to l_{2}}\\ 0 & =c_{l_{1}\to l_{2}}-c_{l_{2}\to l_{3}}\\ & \vdots \\ 0 & =c_{l_{r-1}\to l_{r}}-c_{l_{r}\to j}\\ 1 & =c_{l_{r}\to j} \end{array} \end{array}$$ (11)
  which clearly has one solution where each weight is equal to one.
2. If the right-hand side consists of multiple cycles, then repeatedly using the previous argument we can replace the arcs not containing (*q*_*i*_, *q*_*j*_) with chords. Note, that if the reaction vector corresponding to the chord is already on the right-hand side, then we just have to modify its coefficient. This method decomposes the right-hand side and will leave us with one chordless cycle containing (*q*_*i*_, *q*_*j*_), leading back to the previous case with exactly one solution. Repeating the arc substitutions we can see that each arc becomes a chordless cycle with the reintroduced edges and the arising systems of linear equations have exactly one solution.

The first case above shows that the dimension of the stiochiometric subspace reduces by one for each set of reaction vectors that correspond to edges forming a chordless cycle in |*D*| and the second case shows that is reduced by that exact amount. If *σ* denotes the number of chordless cycles in $\tilde{Q}$, then the deficiency of the reaction network can be computed as $\delta =m-\ell -s=2|\tilde{A}\left|-\right|\tilde{A}\left|-(\right|\tilde{A}\left|-\sigma \right)=\sigma$.

### 3.3 Linear conservation laws

System (6) exhibits conservation in several senses. First of all, we have that

$$\begin{array}{c} \sum_{i=1}^{m}({\dot{n}}_{i}+{\dot{s}}_{i})=0, \end{array}$$ (12)

thus the sum of modeled quantities and free spaces in the system is constant along the trajectories of (6); that is, the function $H:R^{2m}\mapsto R$ defined for $x\in R^{2m}$ as

$$\begin{array}{c} H\left(x\right)=\sum_{i=1}^{2m}x_{i}, \end{array}$$ (13)

is a first integral, where *x*_1_, *x*_2_, …, *x*_*m*_ and *x*_*m*+1_, *x*_*m*+2_, …, *x*_2*m*_ correspond to the variables *n*_1_, *n*_2_, …, *n*_*m*_ and *s*_1_, *s*_2_, …, *s*_*m*_, respectively. Our next observation is that ${\dot{n}}_{i}+{\dot{s}}_{i}=0$ holds for each compartment, thus *c*_*i*_ ≔ *n*_*i*_ + *s*_*i*_ is the constant capacity of compartment *q*_*i*_. Let *c*^(*m*)^ be a vector such that its *i*th coordinate is *c*_*i*_. Substituting *s* = *c*^(*m*)^ − *n* we can rewrite (6) in a reduced state space as

$$\begin{array}{c} \begin{array}{cc} {\dot{n}}_{i} & =\sum_{j\in D_{i}}\;K_{ji}(n,c^{\left(m\right)}-n,t)-\sum_{j\in R_{i}}\;K_{ij}(n,c^{\left(m\right)}-n,t) \end{array} \end{array}$$ (14)

or after an analogous substitution, as

$$\begin{array}{c} \begin{array}{cc} {\dot{s}}_{i} & =-\sum_{j\in D_{i}}\;K_{ji}(c^{\left(m\right)}-s,s,t)+\sum_{j\in R_{i}}\;K_{ij}(c^{\left(m\right)}-s,s,t). \end{array} \end{array}$$ (15)

As a consequence of the preceding observations, the function $\tilde{H}:R^{m}\mapsto R$, defined for $x\in R^{m}$ as

$$\begin{array}{c} \tilde{H}\left(x\right)=\sum_{i=1}^{m}x_{i} \end{array}$$ (16)

is a first integral for (14), in which case each *x*_*i*_ = *n*_*i*_ (and similarly for (15) if each *x*_*i*_ = *s*_*i*_). This shows that while the state space of the decomposed systems is $\tilde{C}:=\left[0,c_{1}\right]\times \left[0,c_{2}\right]\times \ldots \times \left[0,c_{m}\right]$, for a given initial condition $x\left(0\right)\tilde{C}$ the trajectories are contained in the (*m* − 1)-dimensional manifold (hyperplane) defined by

$$\begin{array}{c} \left\{x\in \tilde{C}|\tilde{H}\left(x\right)-\tilde{H}(x\left(0\right))=0\right\}. \end{array}$$ (17)

For a generalized ribosome flow define $c=\sum_{i=1}^{n}c_{i}$ and for *r* ∈ [0, *c*] let $L_{r}\subset \tilde{C}$ be the level set of *H* corresponding to *r*; that is,

$$\begin{array}{c} L_{r}=\left\{a\in \tilde{C}:H\left(a\right)=r\right\}. \end{array}$$ (18)

#### Example 1.1: (generalized) RFMR

As a small example let us consider a Ribosome Flow Model on a Ring (RFMR) [13] with three sites. The underlying compartmental model is given by *D* = (*Q*, *A*), where

$$\begin{array}{c} \begin{array}{cc} Q & =\left\{q_{1},q_{2},q_{2}\right\},\\ A & =\left\{\left(q_{1},q_{2}\right),\left(q_{2},q_{3}\right),\left(q_{3},q_{1}\right)\right\}. \end{array} \end{array}$$ (19)

**Fig 1:**
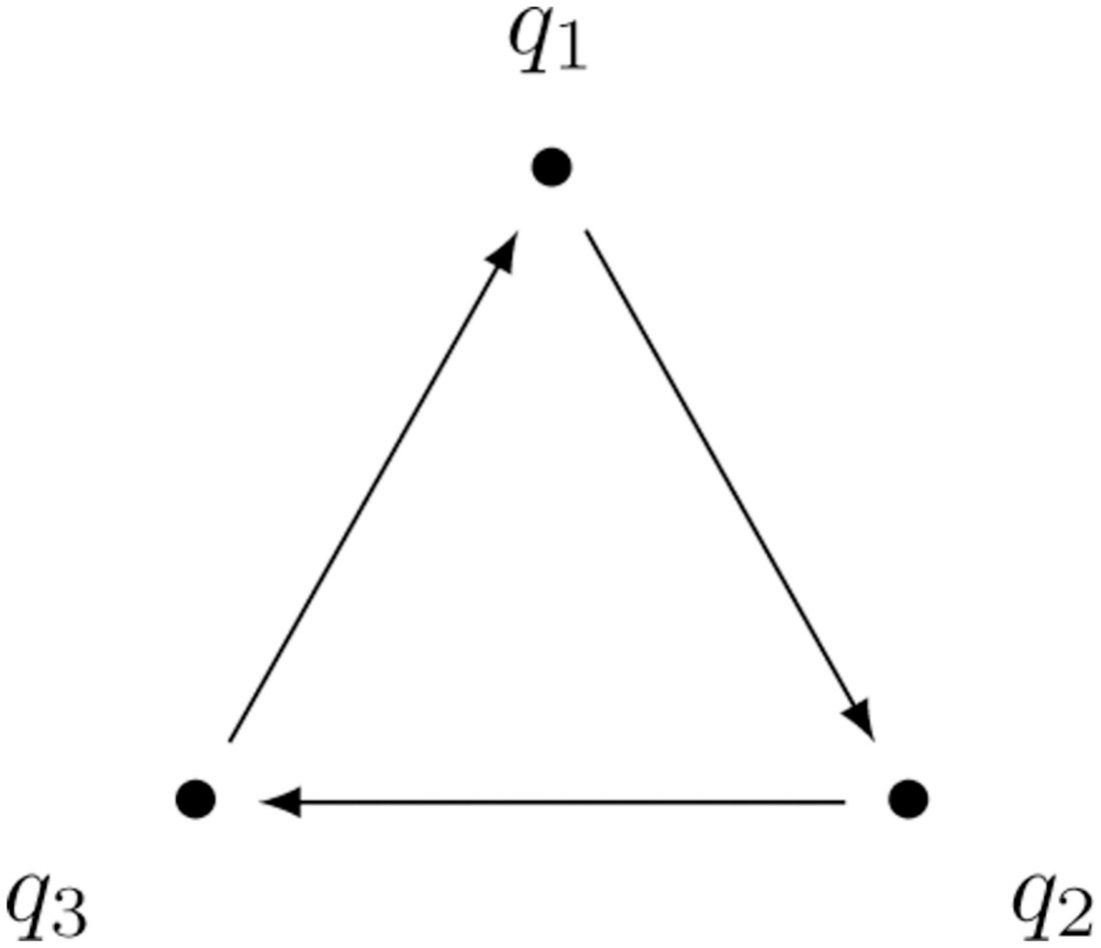
Compartmental graph of a three-dimensional RFMR.

The topology is shown in Fig 1.

The corresponding CRN has the following species and reactions:

$$\begin{array}{c} \begin{array}{cc} & \Sigma =\{N_{1},N_{2},N_{3},S_{1},S_{2},S_{3}\}\\ & R_{1}:N_{1}+S_{2}\overset{K_{12}}{\to }S_{1}+N_{2}\\ & R_{2}:N_{2}+S_{3}\overset{K_{23}}{\to }S_{2}+N_{3}\\ & R_{3}:N_{3}+S_{1}\overset{K_{31}}{\to }S_{3}+N_{1}. \end{array} \end{array}$$ (20)

It is easy to see that, indeed, the reaction graph is not weakly reversible and its deficiency is one. The dynamics of the model in the full state space is given by (6) as

$$\begin{array}{c} \begin{array}{cc} {\dot{n}}_{1} & =K_{31}\left(n,s,t\right)-K_{12}\left(n,s,t\right)\\ {\dot{s}}_{1} & =-K_{31}\left(n,s,t\right)+K_{12}\left(n,s,t\right)\\ {\dot{n}}_{2} & =K_{12}\left(n,s,t\right)-K_{23}\left(n,s,t\right)\\ {\dot{s}}_{2} & =-K_{12}\left(n,s,t\right)+K_{23}\left(n,s,t\right)\\ {\dot{n}}_{3} & =K_{23}\left(n,s,t\right)-K_{31}\left(n,s,t\right)\\ {\dot{s}}_{3} & =-K_{23}\left(n,s,t\right)+K_{31}\left(n,s,t\right) \end{array} \end{array}$$ (21)

which can be rewritten in the reduced state space based on (14) as

$$\begin{array}{c} \begin{array}{cc} {\dot{n}}_{1} & =K_{31}(n,c^{\left(m\right)}-n,t)-K_{12}(n,c^{\left(m\right)}-n,t)\\ {\dot{n}}_{2} & =K_{12}(n,c^{\left(m\right)}-n,t)-K_{23}(n,c^{\left(m\right)}-n,t)\\ {\dot{n}}_{3} & =K_{23}(n,c^{\left(m\right)}-n,t)-K_{31}(n,c^{\left(m\right)}-n,t). \end{array} \end{array}$$ (22)

In a classical RFMR each *c*_*i*_ = 1 and each transition-rate $K_{ij}$ depends only on *n*_*i*_ and *c*_*i*_ − *n*_*i*_ = 1 − *n*_*i*_, and follows the mass-action law. In an RFMR with different site sizes (RFMRD) [15] we allow arbitrary site sizes, in which case the above equation can be written as

$$\begin{array}{c} \begin{array}{cc} {\dot{n}}_{1} & =k_{31}n_{3}\left(c_{1}-n_{1}\right)-k_{12}n_{1}\left(c_{2}-n_{2}\right)\\ {\dot{n}}_{2} & =k_{12}n_{1}\left(c_{2}-n_{2}\right)-k_{23}n_{2}\left(c_{3}-n_{3}\right)\\ {\dot{n}}_{3} & =k_{23}n_{2}\left(c_{3}-n_{3}\right)-k_{31}n_{3}\left(c_{1}-n_{1}\right). \end{array} \end{array}$$ (23)

## 4 Analysis of persistence and stability

In this section we show that systems of the form (6) exhibit various interesting dynamical properties that can be characterized under different assumptions of the transition rate functions. First we will consider time-invariant systems to demonstrate the regularity of equilibria. Then we return to time-varying systems to generalize the results of [27].

### 4.1 Equilibria of time-invariant systems

In this subsection we assume that the $K_{ij}\left(n,s,t\right)$ rate functions are continuously differentiable and only depend on the variables *n*_*i*_ and *s*_*j*_ in a nondecreasing manner; that is, we assume that $K_{ij}\left(n,s,t\right)\equiv K_{ij}\left(n_{i},s_{j}\right)$ for each *i* and *j*. Then the results [27] show that a system of the form (14) is cooperative (the name also highlights the importance of the exclusion of inhibitory phenomena), is (strongly) monotone and each level set *L*_*r*_ contains a unique globally (relative to its level set) asymptotically stable steady state. This implies that the steady states form a linearly ordered set. For *i* = 1, 2, …, *m* let *e*_*i*_: [0, *c*] ↦ [0, *c*_*i*_] denote the *i*th coordinate function of the steady state; that is, let

$$\begin{array}{c} e_{i}\left(r\right)≔\underset{t\to \infty }{\text{log}\;}{\rho (t,n\left(0\right))}_{i} \end{array}$$ (24)

where *n*(0) ∈ *L*_*r*_ is arbitrary and *ρ*(*t*, *n*(0)) denotes the solution at time *t* with *ρ*(0, *n*(0)) = *n*(0). Clearly each *e*_*i*_ is continuous and the monotonicity of the system also shows that each *e*_*i*_ function is strictly increasing; that is, they are differentiable almost everywhere and their derivative are positive.

#### Example 1.2: Equilibria of generalized RFMR

**Fig 2:**
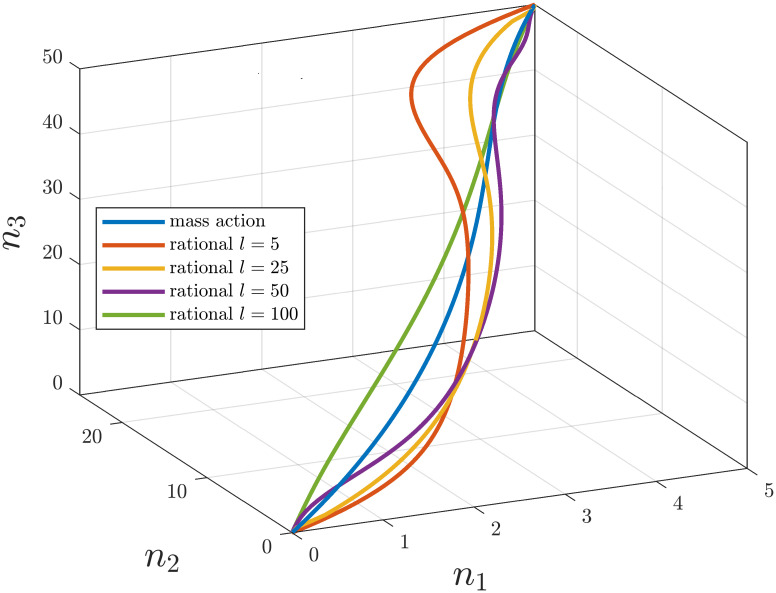
Loci of equilibria of a generalized RFMR as a function of the total number of ribosomes for different *l* saturation parameters.

Let us consider a generalized version of the RFMR in Fig 1. Its time evolution in the reduced state space is given in the form (14) as

$$\begin{array}{c} \begin{array}{cc} & {\dot{n}}_{1}=K_{31}\left(n_{3},c_{1}-n_{1}\right)-K_{12}\left(n_{1},c_{2}-n_{2}\right),\\ & {\dot{n}}_{2}=K_{12}\left(n_{1},c_{2}-n_{2}\right)-K_{23}\left(n_{2},c_{3}-n_{3}\right),\\ & {\dot{n}}_{3}=K_{23}\left(n_{2},c_{3}-n_{3}\right)-K_{31}\left(n_{3},c_{1}-n_{1}\right), \end{array} \end{array}$$ (25)

and for the simulations we set capacities *c*_1_ = 5, *c*_2_ = 25, *c*_3_ = 50. The rate functions in the different cases are assumed to have the form $K_{ij}\left(n_{i},c_{j}-n_{j}\right)=k_{ij}n_{i}\left(c_{j}-n_{j}\right)$ (corresponding to the classical RFMRD) or to be rational functions of the form

$$\begin{array}{c} K_{ij}\left(n_{i},c_{j}-n_{j}\right)=k_{ij}\frac{n_{i}^{3}}{{\left(l+n_{i}\right)}^{3}}\cdot \frac{{\left(c_{j}-n_{j}\right)}^{3}}{{\left(l+c_{j}-n_{j}\right)}^{3}} \end{array}$$

for some *l* > 0 with *k*_12_ = 100, *k*_23_ = 40, *k*_31_ = 60. Fig 2 shows the equilibrium curves for these rate functions with various *l* values.

#### Example 2: Not strongly connected model

Let us consider consider a not strongly connected compartmental model given by *D* = (*Q*, *A*), where

$$\begin{array}{c} \begin{array}{cc} Q & =\left\{q_{1},q_{2},q_{2}\right\},\\ A & =\left\{\left(q_{2},q_{3}\right),\left(q_{3},q_{2}\right),\left(q_{3},q_{1}\right)\right\}. \end{array} \end{array}$$ (26)

**Fig 3:**
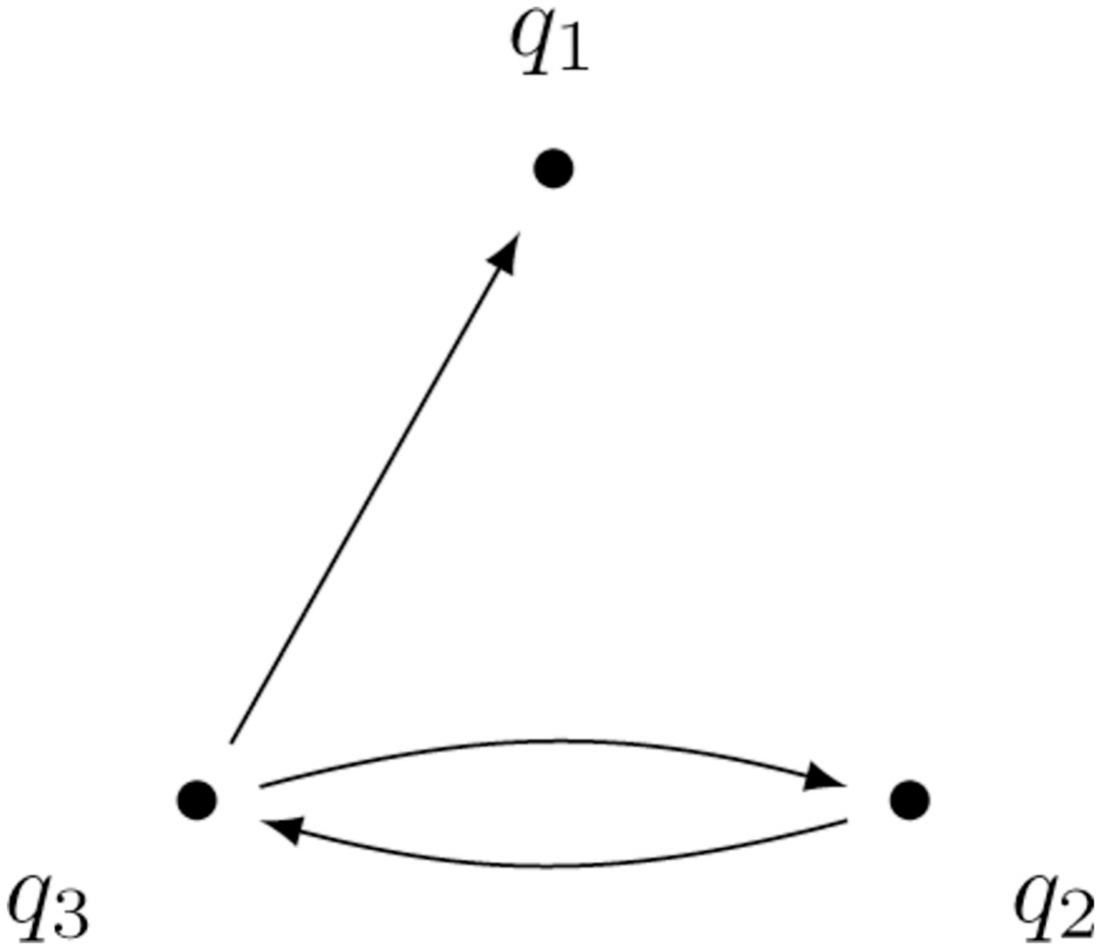
Compartmental graph of a not strongly connected model.

The topology is shown in Fig 3.

The corresponding CRN has the following species and reactions:

$$\begin{array}{c} \begin{array}{cc} & \Sigma =\{N_{1},N_{2},N_{3},S_{1},S_{2},S_{3}\}\\ & R_{1}:N_{2}+S_{3}\overset{K_{23}}{\to }S_{2}+N_{3}\\ & R_{2}:N_{3}+S_{2}\overset{K_{32}}{\to }S_{3}+N_{2}\\ & R_{3}:N_{3}+S_{1}\overset{K_{31}}{\to }S_{3}+N_{1}. \end{array} \end{array}$$ (27)

The dynamics of the system in the reduced state space is given by

$$\begin{array}{c} \begin{array}{cc} {\dot{n}}_{1} & =K_{31}(n_{3},c_{1}-n_{1})\\ {\dot{n}}_{2} & =K_{32}(n_{3},c_{2}-n_{2})-K_{23}(n_{2},c_{3}-n_{3})\\ {\dot{n}}_{3} & =K_{23}(n_{2},c_{3}-n_{3})-K_{32}(n_{3},c_{2}-n_{2})-K_{31}(n_{3},c_{1}-n_{1}). \end{array} \end{array}$$ (28)

Since the compartmental graph is not strongly connected the persistence and stability results of [27] are not applicable. However, empirical results show that the long-time behaviour of the system still exhibits some regularity, which can be divided into two cases base on the initial values of the system:

1. If *r* ≔ *H*(*n*(0)) ≤ *c*_1_, then
  $$\begin{array}{c} \underset{t\to \infty }{\text{lim}\;}n_{2}\left(t\right)=\underset{t\to \infty }{\text{lim}\;}n_{3}\left(t\right)=0\;\text{and}\;\underset{t\to \infty }{\text{lim}\;}n_{1}\left(t\right)=r. \end{array}$$
2. If *r* ≔ *H*(*n*(0)) > *c*_1_, then
  $$\begin{array}{c} \underset{t\to \infty }{\text{lim}\;}n_{1}\left(t\right)=c_{1} \end{array}$$
  and *n*_1_(*t*) and *n*_2_(*t*) will converge to the unique equilibrium on the level set
  $$\begin{array}{c} \left\{\left(n_{2},n_{3}\right)\in \left[0,c_{2}\right]\times \left[0,c_{3}\right]|n_{2}+n_{3}=r-c_{1}\right\} \end{array}$$
  of the reduced compartmental model *D*′ = (*Q*′, *A*′) given by *Q*′ = {*q*_2_, *q*_3_}, $A^{\prime }=\left\{\left(q_{2},q_{3}\right),\left(q_{3},q_{2}\right)\right\}$. Note that since *D*′ is strongly connected, the results of [27] and the above investigation can be applied.

**Fig 4:**
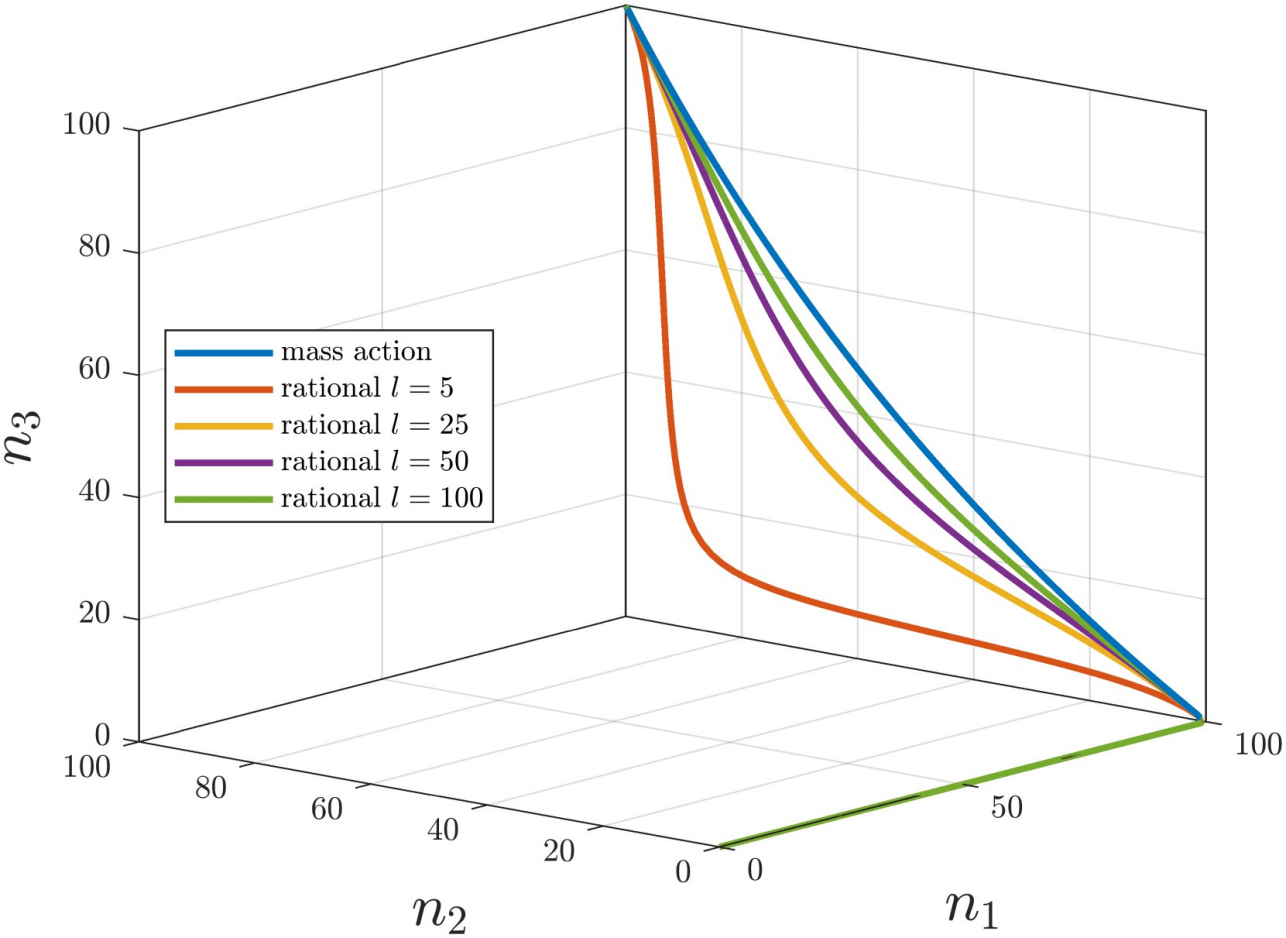
Loci of equilibria of a not strongly connected model as a function of the amount of modeled quantities for different *l* saturation parameters.

For the simulations we set *c*_1_ = *c*_2_ = *c*_3_ = 100. The rate functions in the different cases are assumed to have form $K_{ij}\left(n_{i},c_{j}-n_{j}\right)=k_{ij}n_{i}\left(c_{j}-n_{j}\right)$ (corresponding to mass-action kinetics) or to be rational functions of the form

$$\begin{array}{c} K_{ij}\left(n_{i},c_{j}-n_{j}\right)=k_{ij}\frac{n_{i}}{l+n_{i}}\cdot \frac{c_{j}-n_{j}}{l+c_{j}-n_{j}} \end{array}$$

for some *l* > 0 with *k*_23_ = 15, *k*_32_ = 25, *k*_31_ = 35. Fig 4 shows the equilibrium curves for these rate functions with various *l* values. As described by the above cases we see that until the sum of the initial value exceed the capacity of the *q*_1_ compartment the equilibrium lies on the *n*_1_ axis. After that the equilibrium lies on the plane $\{n_{1}=c_{1}\}\subset R^{3}$ and since *D*′ is strongly connected we have that the coordinate functions of the equilibria *e*_2_(*r*) and *e*_3_(*r*), restricted to the set [*c*_1_, *c*], are continuous and strictly increasing. We note that while the system is not strongly connected it exhibits many similar qualitative properties as strongly connected models. For example, for initial values satisfying *H*(*n*(0)) > *c*_1_ the system is Lyapunov stable as described in [29].

**Remark 4.1.** *The authors hypothesize that the long-time behaviour of a compartmental model with **arbitrary** compartmental structure can be similarly described. Recall that a (compartmental) graph D* = (*Q*, *A*) *can be written as a directed acyclic hypergraph of strongly connected components. The hypergraph will then contain three types of components:*

1. *we call a component trap if it does not have any outgoing edges*,
2. *we call a component source if it does not have any incoming edges*,
3. *we call a component intermediate if it is not a trap and not a source*.

*Based on the initial value and the exact compartmental structure the following phenomena can be observed*:

- *Traps (and only traps) can become full, thus possibly creating new traps*.
- *Sources (and only sources) can become empty, thus possibly creating new sources*.
- *After a sufficient number of traps are filled and sources are emptied, the compartmental graph D is decomposed into isolated strongly connected components; that is, the resulting graph is weakly reversible, in which case the results of* [27] *can be applied*.

*While these observations are elementary and show that the system is stable, the equilibria are clearly non-unique with respect to the total mass of the network and in general it is not straightforward to predict from the initial value which components will fill and empty*.

### 4.2 Persistence

In this subsection we consider time-varying generalized ribosome flows of the form (6) only under mild regularity assumptions described by the following theorem, which is based on the results of [35] but the statements are rephrased to be more aligned with our framework. For the definition of notions related to Petri nets (e.g. siphons) and their exact connection with CRNs we refer to [27, 35].

**Theorem 4.2.** [35] *The dynamics of a CRN of the form* (3) *is persistent if*

*(i) Each siphon of the CRN contains a subset of species which define a positive linear conserved quantity for the dynamics*.
*(ii) There exists a positive linear conserved quantity c*^T^*x* *for the dynamics*.
*(iii) There are nonnegative, continuous functions* ${\underline{K}}_{j}\left(x\right)$ , ${\bar{K}}_{j}\left(x\right)$ *such that*
  *(a) if* $x_{k}>{\tilde{x}}_{k}$ *for each k* ∈ supp(*y*_*j*_), *then* ${\underline{K}}_{j}\left(x\right)>{\underline{K}}_{j}\left(\tilde{x}\right)$ *(and similarly for* ${\bar{K}}_{j}$) *holds for each j* = 1, 2, …, *R*, *and*
  *(ii) for each j* = 1, 2, …, *R*, *for all* $x\in R_{+}^{N}$ *and for all t* ≥ 0 *we have* ${\underline{K}}_{j}\left(x\right)\leq K_{j}\left(x,t\right)\leq {\bar{K}}_{j}\left(x\right)$.

To verify condition (i) we would, in general, need to enumerate all siphons of the CRN, which is well-known to be an NP-hard problem. However, in our recent paper [27] we explicitly characterized the siphons of a CRN assigned to a strongly connected compartmental models in the time-invariant case. However, one can observe that conditions (i) and (ii) of 4.2 are independent of the choice of transition rates and even independent from whether the system is time-invariant or not; that is, our results, formulated in the following theorem, hold for time-varying compartmental systems as well.

**Theorem 4.3.** [27, *Corollary 4.6*] *A siphon in the Petri net of a strongly connected compartmental graph either contains the vertices N*_*i*_ *and S*_*i*_ *corresponding to the same compartment q*_*i*_, *or it contains all the vertices N*_1_, *N*_2_, …, *N*_*m*_ *or S*_1_, *S*_2_, …, *S*_*m*_.

Then the conclusions of Section 3.3 show that conditions (i) and (ii) are satisfied by virtue of the first integrals (16) and (13), respectively.

It is not straightforward to determine exactly what types of reaction rates satisfy condition (iii). For the sake of specificity, we characterize a class of reaction rates of special interest which can be written in the following form

$$\begin{array}{c} K_{ij}\left(n,s,t\right)=k_{ij}\left(t\right)\frac{\theta_{i}\left(n_{i}\right)\nu_{j}\left(s_{j}\right)}{1+\Psi_{ij}\left(n,s\right)} \end{array}$$ (29)

where we assume that the transformations $\theta_{i},\nu_{j}\in C^{1}\left(R\right)$ are nondecreasing, have *θ*_*i*_(0) = *ν*_*j*_(0) = 0 and satisfy $\int_{0}^{1}\left|\text{log}\;\theta_{i}\left(r\right)\right|dr<\infty$ and $\int_{0}^{1}\left|\text{log}\;\nu_{j}\left(r\right)\right|dr<\infty$ for each *i*, *j* = 1, 2, …, *m*. We also assume that the functions Ψ_*ij*_ take the form

$$\begin{array}{c} \Psi_{ij}\left(n,s\right)=\sum \alpha_{r^{\left(1\right)},r^{\left(2\right)}}\prod_{l=1}^{m}\theta_{l}^{r_{l}^{\left(1\right)}}\left(n_{l}\right)\nu_{l}^{r_{l}^{\left(2\right)}}\left(s_{l}\right) \end{array}$$ (30)

where $r^{\left(1\right)},r^{\left(2\right)}\in N^{m}$ and $\alpha_{r^{\left(1\right)},r^{\left(2\right)}}\in {\bar{R}}_{+}$. We further assume that for *k*_*ij*_(*t*) there exist ${\underline{k}}_{ij},{\bar{k}}_{ij}>0$ such that ${\underline{k}}_{ij}\leq k_{ij}\left(t\right)\leq {\bar{k}}_{ij}$ for all *t* ≥ 0. In this case we have

$$\begin{array}{c} {\underline{K}}_{ij}\left(n_{i},s_{j}\right)≔\frac{{\underline{k}}_{ij}}{1+\Psi_{ij}\left(c^{\left(m\right)},c^{\left(m\right)}\right)}\theta_{i}\left(n_{i}\right)\nu_{j}\left(s_{j}\right)\leq K_{ij}\left(n_{i},s_{j},t\right)\leq {\bar{k}}_{ij}\theta_{i}\left(n_{i}\right)\nu_{j}\left(s_{j}\right)=:{\bar{K}}_{ij}\left(n_{i},s_{j}\right) \end{array}$$ (31)

which are clearly monotonous in the sense of Theorem 4.2, and thus condition (i) is satisfied and the system is persistent.

**Remark 4.4.** *The above investigation and, in particular, condition (iii) of Theorem 4.2 shows that Lemmata 5.1, 5.2 and Remark 5.3 of* [27] *can be modified to the time-varying case; that is, for a system of the form* (6) *with strongly connected compartmental graph and reaction rates of the form* (29), *for each* *τ* > 0 *there exists ϵ*(*τ*) > 0 with *ϵ*(*τ*) → 0 *as τ* → 0 *such that n*_*i*_(*t*), *s*_*i*_(*t*) ∈ [*ϵ*, *c*_*i*_ − *ϵ*] *holds for each i* = 1, 2, …, *m and t* ≥ *τ*.

The denominator of (29) contains positive terms which can be interpreted as the inhibitory effect of other species, and the time-varying coefficient *k*_*ij*_(*t*) introduces the dependence of the system parameters on various factors such as temperature or the dynamical behaviour of other species that are not explicitly modeled as state variables. This class of rate functions contains many well-known examples, demonstrating the range and flexibility of reaction rates of the above form:

1. Setting each *θ*_*i*_(*n*_*i*_) = *n*_*i*_ and *ν*_*j*_(*s*_*j*_) = *s*_*j*_ and Ψ_*ij*_(*n*, *s*) = 0 we obtain the case of classical mass-action kinetics with time-varying rate coefficients: $K_{ij}\left(n,s,t\right)=k_{ij}\left(t\right)n_{i}s_{j}$.
2. Setting each *θ*_*i*_(*n*_*i*_) = *n*_*i*_ and *ν*_*j*_(*s*_*j*_) = *s*_*j*_ and Ψ_*ij*_(*n*, *s*) = *l*^2^ − 1 + *ln*_*i*_ + *ls*_*j*_ + *n*_*i*_*s*_*j*_ for some *l* > 0 yields
  $$\begin{array}{c} K_{ij}\left(n,s,t\right)=k_{ij}\left(t\right)\frac{n_{i}s_{j}}{\left(l+n_{i}\right)\left(l+s_{j}\right)} \end{array}$$ (32)
  corresponding to simple saturating kinetics described by the Monod equation.
3. The previous example can also be obtained by setting $\theta_{i}\left(n_{i}\right)=\frac{n_{i}}{l+n_{i}}$ and $\nu_{j}\left(s_{j}\right)=\frac{s_{j}}{l+s_{j}}$ and Ψ_*ij*_(*n*, *s*) = 0, showing that (29) is not unique. Notice however, that for fixed *θ*_*i*_, *ν*_*j*_ transformations the function Ψ_*ij*_, and thus the fraction itself, is unique.
4. Setting each $\theta_{i}\left(n_{i}\right)=\frac{n_{i}^{L}}{l+n_{i}^{L}}$ and $\nu_{j}\left(s_{j}\right)=\frac{s_{j}^{L}}{l+s_{j}^{L}}$ for some *l* > 0 yields the classical Hill kinetics.

#### Example 1.3: Time-varying generalized RFMR

Let us again consider a generalized version of the RFMR from Fig 1. For this example we set *c*_1_ = *c*_2_ = *c*_3_ = 100, *l* = 100 and

$$\begin{array}{c} \begin{array}{cc} K_{12}\left(n_{1},c_{2}-n_{2},t\right) & =k_{12}\left(t\right)\frac{n_{1}\left(c_{2}-n_{2}\right)}{\left(l+n_{1}\right)\left(l+c_{2}-n_{2}\right)},\\ K_{23}\left(n_{2},c_{3}-n_{3},t\right) & =k_{23}\left(t\right)\frac{n_{2}\left(c_{3}-n_{3}\right)}{\left(l+n_{2}\right)\left(l+c_{3}-n_{3}\right)},\\ K_{31}\left(n_{3},c_{1}-n_{1},t\right) & =k_{31}\left(t\right)\frac{n_{3}\left(c_{1}-n_{1}\right)}{\left(l+n_{3}\right)\left(l+c_{1}-n_{1}\right)}, \end{array} \end{array}$$ (33)

where the coefficient functions are considered to be exponentially decaying perturbations of the nominal values

$$\begin{array}{c} {\bar{k}}_{12}=40\;{\bar{k}}_{23}=25\;{\bar{k}}_{31}=50 \end{array}$$ (34)

of the form

$$\begin{array}{c} k_{12}\left(t\right)={\bar{k}}_{12}(1+e^{-\frac{3t}{100}})\;k_{23}(1+e^{-\frac{5t}{100}})\;k_{31}(1+e^{-\frac{2t}{100}}). \end{array}$$ (35)

**Fig 5:**
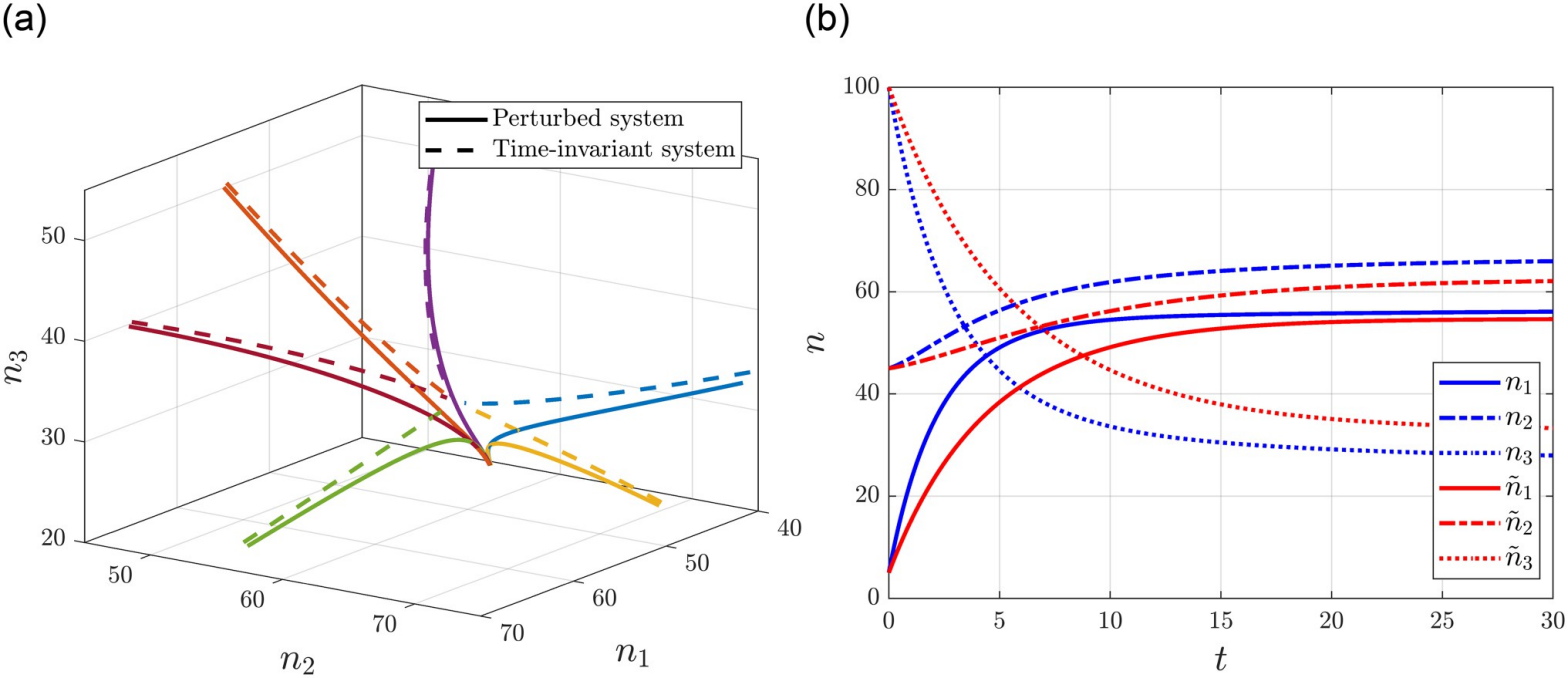
Trajectories and time evolution of a generalized time-varying RFMR with decaying time dependence. (**a**) Phase portrait of the system. (**b**) Time evolution of state variables.

As a comparison let us consider the solution $\tilde{n}\left(t\right)$ of the time-invariant system with the above nominal values. Fig 5a shows the phase portrait of the perturbed and the original systems starting from various initial conditions with *H*(*n*(0)) = 150. Fig 5b shows the time evolution of the state variables with *n*(0) = [5 45 100]^T^, where the state variables of the perturbed and the time-invariant system are depicted with blue lines and red lines, respectively. We can observe that since the time dependent terms are exponentially decaying and both systems evolve on the same linear manifold, the systems tend to the same equilibrium, as expected.

### 4.3 Stability of the solutions for periodic transition rates

In this section we investigate the periodic behaviour of the generalized ribosome flows based on the ideas of [36]. Let us consider a generalized ribosome flow in the reduced state space of the form (14) with transition rates of the form (29) and assume that the transition functions are $C^{1}$ and periodic with the same period (but having possibly different phases). Write (14) as $\dot{n}=F\left(t,n\right)$ and assume that the right-hand side satisfies the following monotonicity condition: *F*_*i*_(*t*, *x*) ≤ *F*_*i*_(*t*, *y*) for any two distinct points $x,y\in \tilde{C}$ such that *x*_*i*_ = *y*_*i*_ and *x*_*j*_ ≤ *y*_*j*_ for *j* ≠ *i*. This condition is satisfied if, for example, the transition rates are such that Ψ_*ij*_ ≡ 0; that is, if there are no inhibitory phenomena. Then the system phase locks (or entrains) with the periodic excitations.

**Theorem 4.5.** *Consider a system of the form* (14) *satisfying the above monotonicity assumption, where each* $K_{ij}\left(t\right)$ *is periodic with a common period T*. *Then for each r* ∈ [0, *c*] *there exists a unique periodic function* $\phi_{r}:{\bar{R}}_{+}:\mapsto \tilde{C}$ *with period T such that for all a* ∈ *L*_*r*_ *we have that*

$$\begin{array}{c} \underset{t\to \infty }{\text{lim}\;}∥{\rho \left(t,a\right)-\phi_{r}\left(t\right)∥}_{L^{1}}=0. \end{array}$$ (36)

*Proof*. The properties of the rate functions and the fact that ∇*H* is positive implies the result via [37, 38].

**Remark 4.6.** *Since, in some sense, time-invariant systems can be seen as periodic, the stability result* [27, *Proposition 5.5] is a special case of the above theorem, where ϕ*_*r*_ *is reduced to a single point of the manifold L*_*r*_.

#### Example 1.4: Entrainment of generalized RFMR

**Fig 6:**
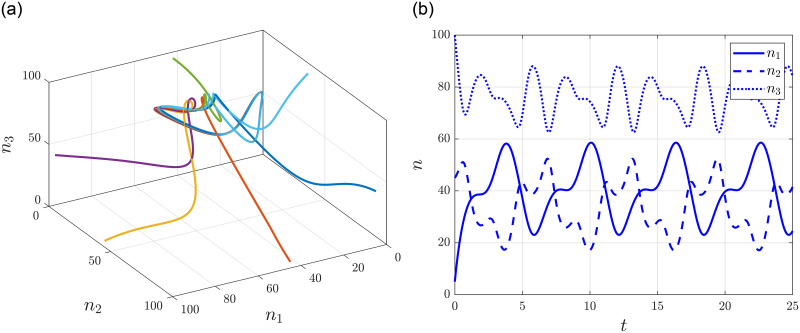
Entrainment of a generalized RFMR with periodic transition rates. **(a)** Phase portrait of the system. **(b)** Time evolution of state variables.

Let us again consider a generalized version of the RFMR from Fig 1. For this example we set *c*_1_ = *c*_2_ = *c*_3_ = 100 and

$$\begin{array}{c} \begin{array}{cc} K_{12}\left(n_{1},c_{2}-n_{2},t\right) & =100(3+2\;\text{cos}\;\left(t+0.5\right))\frac{n_{1}\left(c_{2}-n_{2}\right)}{\left(l+n_{1}\right)\left(l+c_{2}-n_{2}\right)},\\ K_{23}\left(n_{2},c_{3}-n_{3},t\right) & =100(7+5\;\text{sin}\;\left(3t-2.5\right))\frac{n_{2}\left(c_{3}-n_{3}\right)}{\left(l+n_{2}\right)\left(l+c_{3}-n_{3}\right)},\\ K_{31}\left(n_{3},c_{1}-n_{1},t\right) & =100(2+\text{cos}\;\left(2t-1\right))\frac{n_{3}\left(c_{1}-n_{1}\right)}{\left(l+n_{3}\right)\left(l+c_{1}-n_{1}\right)}, \end{array} \end{array}$$ (37)

which clearly have the same period *T* = 2*π*. Fig 6a and 6b show the phase portrait of the system starting from various initial conditions with *l* = 100, *H*(*n*(0)) = 150 and the time evolution of the state variables with *n*(0) = [5 45 100]^T^, respectively.

## 5 Lyapunov stability analysis

In this section we show that generalized ribosome flows with reaction rate functions of the form (29) with piecewise locally Lipschitz *k*_*ij*_(*t*) coefficients satisfy a certain notion of robustness to the changes in the time-varying rate functions that can be traced back to the input-to-state stability of rate-controlled biochemical networks thoroughly investigated in [22]. The main difficulty in applying these results lies in the aforementioned fact that the CRN assigned to a compartmental model is generally not weakly reversible and its deficiency is generally not zero (see, Theorem 3.1) even if the compartmental topology is strongly connected. In order to circumvent this, we will perform a model reduction and rewrite (14) by factoring out appropriate terms. Let us first recall the most important notions and results of [22].

Consider the system corresponding to a CRN with *R* reactions

$$\begin{array}{c} \dot{x}=f\left(x,u\right)=\sum_{i=1}^{R}\sum_{j=1}^{R}u_{ij}\left(t\right)\prod_{l=1}^{n}\theta_{i}^{y_{ij}}\left(x_{i}\right)\left[y_{i}-y_{j}\right], \end{array}$$ (38)

where the nonnegative functions *u*_*ij*_ are piecewise locally Lipschitz with a finite number of discontinuities and the stoichiometric coefficient vectors *y*_*i*_, *y*_*j*_ are as described in 2.2. Motivated by control designs for ribosome flow models [39] we introduce such time dependence not only to handle some uncertainty originating from fluctuating external factors but to measure the robustness of the system to certain control inputs.

In this section, however, we restrict the conditions on the transformation functions $\theta_{i}:{\bar{R}}_{+}\mapsto \left[0,\infty \right)$. Namely, we assume that

(a) *θ*_*i*_ is real analytic,
(b) *θ*_*i*_(0) = 0,
(c) $\int_{0}^{1}\left|\text{log}\;\theta_{i}\left(r\right)\right|dr<\infty$

(d) *θ*_*i*_ is strictly increasing and onto the set [0, *σ*_*i*_) for some *σ*_*i*_ ∈ [0, ∞),
(e) ${\text{lim}\;}_{t\to \text{log}\;\sigma_{i}}\int_{a}^{t}\rho_{i}^{-1}\left(r\right)dr-pt=\infty$ for any *a* < log *σ*_*i*_ and any constant *p* > 0, where *ρ*_*i*_ = log *θ*_*i*_.

Before continuing with the definitions, we consider the case when *u*(*t*) is a constant matrix *A*. We assume that *A* has nonnegative entries and is irreducible; that is, the underlying reaction graph is strongly connected. We denote the set of such *A* matrices as A. Then the equilibria of $\dot{x}=f\left(x,A\right)$ can be divided into the sets of boundary equilibria and positive equilibria:

$$\begin{array}{c} \begin{array}{cc} & E_{0}=\left\{x\in \partial {\bar{R}}_{+}^{n}|f\left(x,A\right)=0\right\},\\ & E_{A,+}=\left\{x\in R_{+}^{n}|f\left(x,A\right)=0\right\}. \end{array} \end{array}$$ (39)

Then, the result [22, Theorem 2.1] (and also [40, Theorem 2]) shows that if there are no boundary equilibria in any positive class, then each positive class contains a unique globally (relative to the positive class) asymptotically stable positive equilibrium. Denote the unique positive equilibrium in the same class as *x*_0_ as $\bar{x}\left(x_{0},A\right)$ and notice that $E_{A,+}=\{\bar{x}\left(x_{0},A\right)|x_{0}\in R_{+}^{n}\}$. Finally, denote

$$\begin{array}{c} E=\underset{A\in A}{⋃}E_{A,+}. \end{array}$$ (40)

**Definition 5.1** *We define the following function classes:*

*(i) A function* $\alpha :{\bar{R}}_{+}\mapsto {\bar{R}}_{+}$ *is said to be of class* K *if it is continuous, strictly increasing and has α*(0) = 0.
*(ii) The subset of unbounded functions of class* K *are denoted by* $K_{\infty }$ .
*(iii) A function* $\beta :{\bar{R}}_{+}\times {\bar{R}}_{+}$ *is said to be of class* KL if *β*(., *t*) *is of class* K *for all t* ≥ 0 *and β*(*r*,.) *is strictly decreasing to zero for all r* > 0.

We consider nonnegative time-varying inputs such that at any time instant the reaction graph is strongly connected; that is, the input-value set U is a subset of A. Furthermore, let ‖.‖_2_ denote the spectral norm induced by the Euclidian norm and for $u:{\bar{R}}_{+}\mapsto U$ define

$$\begin{array}{c} {∥u∥}_{U}=\underset{t\in [0,\infty )}{\text{ess}\;\text{sup}\;}{∥u(t)∥}_{2}. \end{array}$$ (41)

**Definition 5.2.** *A system* $\dot{x}=f\left(x,u\right)$ *is uniformly input-to-state stable (ISS) with input-value set* U *if for every compact set* P⊂E *and every compact set* $F\subset {\bar{R}}_{+}^{n}$ *containing P*, *there exist functions β* = *β*_*P*_ *of class* KL *and ϕ* = *ϕ*_*P*_ *of class* $K_{\infty }$ *such that, for every* ${\bar{x}}_{o}\in P\cap E_{u_{0},+}$ *for some* $u_{0}\in U$ *we have that*

$$\begin{array}{c} ∥x\left(t\right)-{\bar{x}}_{0}∥\leq \beta (∥x_{0}-{\bar{x}}_{0}∥,t)+\phi ({∥u-u_{0}∥}_{U}) \end{array}$$ (42)

*holds for each* $u:{\bar{R}}_{+}\mapsto U$ *input and every initial condition* $x_{0}\in F\cap S_{{\bar{x}}_{0}}$ *and for all t* ≥ 0 *such that x*(*s*) ∈ *F for s* ∈ [0, *t*].

According to the above definition we say that a system is ISS if it is globally asymptotically stable in the absence of external inputs and if its trajectories are bounded by an appropriate function of the input. In some sense this definition is intended to capture the idea of “bounded input bounded output” stability, since for bounded *u* input (*u* − *u*_0_ to be more precise) the trajectories will remain in a ball and, in fact, approach the ball $\phi ({∥u-u_{0}∥}_{U})$ as *t* increases [41].

We assume that there exists a uniform lower bound on the parameters; that is, we consider input-value sets of the form

$$\begin{array}{c} A⊃U_{\epsilon }=\left\{u\in A|u_{ij}\left(t\right)\geq \epsilon \;\forall t\geq 0\text{,}\;\text{or}\;u_{ij}\left(t\right)=0\;\forall t\geq 0\right\}. \end{array}$$ (43)

We also recall that the input functions are piecewise locally Lipschitz in time with a finite number of discontinuities, thus we introduce

$$\begin{array}{c} W=\{w:{\bar{R}}_{+}\mapsto U_{\epsilon }|w\;\text{is}\;\text{piecewise}\;\text{locally}\;\text{Lipschitz}\}. \end{array}$$ (44)

Then the main Theorem of [22] states:

**Theorem 5.3.** *Consider the system* (38) *with and suppose that is is mass-conservative; that is, there exists* $v\in R_{+}^{n}$ *such that* *v*^T^*f*(*x*, *u*) = 0 *for all* $x\in {\bar{R}}_{+}^{n}$ *and* u∈A. *Then the system with input maps* u∈W *is uniformly ISS with input-value set* $U_{\in }$.

The proof relies on the candidate ISS-Lyapunov function (for the definition of which and for the exact connection with ISS stability we refer to [22])

$$\begin{array}{c} V\left(x,\bar{x}\right)=\sum_{i=1}^{n}\int_{{\bar{x}}_{i}}^{x_{i}}(\text{log}\;\theta_{i}\left(r\right)-\text{log}\;\theta_{i}\left({\bar{x}}_{i}\right))dr \end{array}$$ (45)

which, for mass-action systems, yields the classical entropy-like Lyapunov function well-known from the theory of chemical reaction networks, see (65). We note that $V(x,\bar{x})$ is uniquely determined by the *θ*_*i*_ functions and does not depend explicitly on the reaction/compartmental structure or the time-varying *u*_*ij*_(*t*) functions; that is, it is universal in the sense of [42].

**Remark 5.4.** *We note that the assumption that the compartmental graph (and thus the reaction graph of the factored model) is strongly connected is purely technical. For time-invariant systems it simply ensures that the unique equilibrium on each level set of the first integral is positive (except for the trivial case of an empty network of course). In fact, in some cases the initial values of the network can ensure the positivity of the equilibrium even for not strongly connected systems (see Example 2 and* [29] *for more details), in which case the above Lyapunov function can be applied*.

### 5.1 Factorization of the transition rates

Let us consider a generalized ribosome flow in the reduced state space of the form (14), in this case given by

$$\begin{array}{c} \begin{array}{cc} {\dot{n}}_{i} & =\sum_{j\in D_{i}}K_{ji}\left(n,c-n,t\right)-\sum_{j\in R_{i}}K_{ij}\left(n,c-n,t\right)\\ & =\sum_{j\in D_{i}}k_{ji}\left(t\right)\frac{\theta_{j}\left(n_{j}\right)\nu_{i}\left(c_{i}-n_{i}\right)}{1+\Psi_{ji}\left(n,c^{\left(m\right)}-n\right)}-\sum_{j\in R_{i}}k_{ij}\left(t\right)\frac{\theta_{i}\left(n_{i}\right)\nu_{j}\left(c_{j}-n_{j}\right)}{1+\Psi_{ij}\left(n,c^{\left(m\right)}-n\right)}. \end{array} \end{array}$$ (46)

Notice that we can naturally factor some terms of the transition rates into the time-varying coefficient as

$$\begin{array}{c} k_{ij}\left(t\right)\frac{\theta_{i}\left(n_{i}\right)\nu_{j}\left(c_{j}-n_{j}\right)}{1+\Psi_{ij}\left(n,c^{\left(m\right)}-n\right)}=\frac{k_{ij}\left(t\right)\nu_{j}\left(c_{j}-n_{j}\right)}{1+\Psi_{ij}\left(n,c^{\left(m\right)}-c\right)}\theta_{i}\left(n_{i}\right)=:{\tilde{k}}_{ij}\left(t\right)\theta_{i}\left(n_{i}\right). \end{array}$$ (47)

Then (46) can be rewritten as

$$\begin{array}{c} {\dot{n}}_{i}=\sum_{j\in D_{i}}{\tilde{k}}_{ji}\left(t\right)\theta_{j}\left(n_{j}\right)-\sum_{j\in R_{i}}{\tilde{k}}_{ij}\left(t\right)\theta_{i}\left(n_{i}\right). \end{array}$$ (48)

This equation can be clearly embedded into the class of strongly connected systems of the form (38), since the reaction graph of (48) consists of species Σ = {*N*_1_, *N*_2_, …, *N*_*m*_}, has the *m* × *m* identity matrix as its stoichiometric matrix and for each transition (*q*_*i*_, *q*_*j*_) ∈ *A* we assign a reaction of the form

$$\begin{array}{c} N_{i}\overset{{\tilde{K}}_{ij}\left(t\right)}{\to }N_{j}, \end{array}$$ (49)

and thus the system of differential equations can be written as

$$\begin{array}{c} \dot{n}=I{\tilde{A}}_{k}\left(t\right)\theta \left(n\right) \end{array}$$ (50)

where the elements of ${\tilde{A}}_{k}$ are given by

$$\begin{array}{c} {}[{\tilde{A}}_{k}{\left(t\right)]}_{ij}=\{\begin{array}{cc} -\sum_{l\in R_{i}}{\tilde{k}}_{il}\left(t\right)\; & \text{if}\;i=j,\\ {\tilde{k}}_{ji}\left(t\right)\; & \text{if}\;j\in D_{i},\\ 0\; & \text{otherwise}. \end{array} \end{array}$$ (51)

Note that the fractions $\frac{\nu_{j}\left(c_{j}-n_{j}\right)}{1+\Psi_{ij}\left(n,c^{\left(m\right)}-n\right)}$ are differentiable (and thus Lipschitz) and each *k*_*ij*_(*t*) is piecewise locally Lipschitz, hence each ${\tilde{k}}_{ij}\left(t\right)$ is piecewise locally Lipschitz. This shows that generalized ribosome flows can be embedded into the class of rate-controlled biochemical networks described in [22] in a way that preserves the compartmental structure; that is, the reaction graph of (50) is topologically isomorph to the compartmental graph. In particular if the compartmental model is strongly connected, then the reaction graph of the reduced system (50) is strongly connected as well. Furthermore, combining the persistence of the system with Remark 4.4 we find that ${\tilde{A}}_{k}\in W$, and thus Theorem 5.3 ensures input-to-state stability.

### 5.2 Quasi-LTV factorization

A classical argument shows that the model reduction above can result in a Linear Time-Varying (LTV) system [6]. Consider an $F\left(x\right)\in C^{k}\left(R\right)$ nonnegative function such that *F*(0) = 0, where *k* ≥ 1. Then for the function *F*(*rx*) we have

$$\frac{\text{d}F\left(rx\right)}{\text{d}r}\begin{array}{c} =xF^{\prime }\left(rx\right) \end{array}$$ (52)

and thus

$$\begin{array}{c} F\left(x\right)-F\left(0\right)=x\int_{0}^{1}F^{\prime }\left(rx\right)dr=xf\left(x\right) \end{array}$$ (53)

and since *F*(0) = 0, we find that *F*(*x*) = *xf*(*x*). Note, that the calculation also shows that $f\in C^{k-1}\left(R\right)$. Since *θ*_*i*_ is real analytic we have that $\theta_{i}\left(n_{i}\right)={\hat{\theta }}_{i}\left(n_{i}\right)n_{i}$ for some ${\hat{\theta }}_{i}$ real analytic function. Then (48) can be rewritten as

$$\begin{array}{c} {\dot{n}}_{i}=\sum_{j\in D_{i}}{\hat{k}}_{ji}\left(t\right)n_{j}-\sum_{j\in R_{i}}{\hat{k}}_{ij}\left(t\right)n_{i} \end{array}$$ (54)

where

$$\begin{array}{c} {\hat{k}}_{ij}\left(t\right)=\frac{k_{ij}\left(t\right){\hat{\theta }}_{i}\left(n_{i}\right)\nu_{j}\left(c_{j}-n_{j}\right)}{1+\Psi_{ij}\left(n,c^{\left(m\right)}-n\right)}. \end{array}$$ (55)

Similarly as before, the reaction graph of (54) consists of species Σ = {*N*_1_, *N*_2_, …, *N*_*m*_}, has the *m* × *m* identity matrix as its stoichiometric matrix and for each transition (*q*_*i*_, *q*_*j*_) ∈ *A* we assign a reaction of the form

$$\begin{array}{c} N_{i}\overset{{\hat{K}}_{ij}\left(t\right)}{\to }N_{j}, \end{array}$$ (56)

and thus the system of differential equations can be written as

$$\begin{array}{c} \dot{n}=I{\hat{A}}_{k}\left(t\right)n \end{array}$$ (57)

where the elements of ${\hat{A}}_{k}$ are given by

$$\begin{array}{c} {}[{\hat{A}}_{k}{\left(t\right)]}_{ij}=\{\begin{array}{cc} -\sum_{l\in R_{i}}{\hat{k}}_{il}\left(t\right)\; & \text{if}\;i=j,\\ {\hat{k}}_{ji}\left(t\right)\; & \text{if}\;j\in D_{i},\\ 0\; & \text{otherwise}. \end{array} \end{array}$$ (58)

Again, each ${\hat{k}}_{ij}\left(t\right)$ is piecewise locally Lipschitz, thus for strongly connected compartmental models Theorem 5.3 ensures input-to-state stability via Remark 4.4.

### 5.3 Factorization of Monod kinetics

Let us consider a generalized version of the RFMR in Fig 1 with rational rate functions corresponding to Monod kinetics of the form

$$\begin{array}{c} \begin{array}{cc} {\dot{n}}_{1} & =k_{31}\left(t\right)\frac{n_{3}}{l+n_{3}}\frac{c_{1}-n_{1}}{l+c_{1}-n_{1}}-k_{12}\left(t\right)\frac{n_{1}}{l+n_{1}}\frac{c_{2}-n_{2}}{l+c_{2}-n_{2}}\\ {\dot{n}}_{2} & =k_{12}\left(t\right)\frac{n_{1}}{l+n_{1}}\frac{c_{2}-n_{2}}{l+c_{2}-n_{2}}-k_{23}\left(t\right)\frac{n_{2}}{l+n_{2}}\frac{c_{3}-n_{3}}{l+c_{3}-n_{3}}\\ {\dot{n}}_{3} & =k_{23}\left(t\right)\frac{n_{2}}{l+n_{2}}\frac{c_{3}-n_{3}}{l+c_{3}-n_{3}}-k_{31}\left(t\right)\frac{n_{3}}{l+n_{3}}\frac{c_{1}-n_{1}}{l+c_{1}-n_{1}} \end{array} \end{array}$$ (59)

for some *l* > 0. As discussed before, the corresponding CRN is not strongly connected. However, using the functions

$$\begin{array}{c} {\tilde{k}}_{31}\left(t\right)=k_{31}\left(t\right)\frac{c_{1}-n_{1}}{l+c_{1}-n_{1}}\;{\tilde{k}}_{12}\left(t\right)=k_{12}\left(t\right)\frac{c_{2}-n_{2}}{l+c_{2}-n_{2}}\;{\tilde{k}}_{23}\left(t\right)=k_{23}\left(t\right)\frac{c_{3}-n_{3}}{l+c_{3}-n_{3}} \end{array}$$ (60)

we can to rewrite (59) as

$$\begin{array}{c} \begin{array}{cc} {\dot{n}}_{1} & ={\tilde{k}}_{31}\left(t\right)\frac{n_{3}}{l+n_{3}}-{\tilde{k}}_{12}\left(t\right)\frac{n_{1}}{l+n_{1}}\\ {\dot{n}}_{2} & ={\tilde{k}}_{12}\left(t\right)\frac{n_{1}}{l+n_{1}}-{\tilde{k}}_{23}\left(t\right)\frac{n_{2}}{l+n_{2}}\\ {\dot{n}}_{3} & ={\tilde{k}}_{23}\left(t\right)\frac{n_{2}}{l+n_{2}}-{\tilde{k}}_{31}\left(t\right)\frac{n_{3}}{l+n_{3}}. \end{array} \end{array}$$ (61)

Then the CRN corresponding to (64) has the following species and reactions:

$$\begin{array}{c} \begin{array}{cc} & \Sigma =\left\{N_{1},N_{2},N_{3}\right\}\\ & R_{1}:N_{1}\overset{{\tilde{k}}_{12}}{\to }N_{2}\\ & R_{2}:N_{2}\overset{{\tilde{k}}_{23}}{\to }N_{3}\\ & R_{3}:N_{3}\overset{{\tilde{k}}_{31}}{\to }N_{1}. \end{array} \end{array}$$ (62)

which is strongly connected and isomorph to the compartmental model in Fig 1. We arrive at the same conclusion if we instead use the functions

$$\begin{array}{cc} {\hat{k}}_{31}\left(t\right) & =k_{31}\left(t\right)\frac{1}{l+n_{3}}\frac{c_{1}-n_{1}}{l+c_{1}-n_{1}}\\ {\hat{k}}_{12}\left(t\right) & =k_{12}\left(t\right)\frac{1}{l+n_{1}}\frac{c_{2}-n_{2}}{l+c_{2}-n_{2}}\\ {\hat{k}}_{23}\left(t\right) & =k_{23}\left(t\right)\frac{1}{l+n_{2}}\frac{c_{3}-n_{3}}{l+c_{3}-n_{3}} \end{array}$$ (63)

to rewrite (59) as

$$\begin{array}{cc} {\dot{n}}_{1} & ={\hat{k}}_{31}\left(t\right)n_{3}-{\hat{k}}_{12}\left(t\right)n_{1}\\ {\dot{n}}_{2} & ={\hat{k}}_{12}\left(t\right)n_{1}-{\hat{k}}_{23}\left(t\right)n_{2}\\ {\dot{n}}_{3} & ={\hat{k}}_{23}\left(t\right)n_{2}-{\hat{k}}_{31}\left(t\right)n_{3}. \end{array}$$ (64)

Note that the quasi-LTV factorization might be more complicated in some cases, but the construction described in Section 5.2 guarantees its existence.

### 5.4 Induced family of Lyapunov functions

The above investigation demonstrates that generalized ribosome flows can be embedded into rate-controlled biochemical networks in at least two different ways, where each embedding induces a different Lyapunov function of the form (45). Thus, in general, we may use at least two different Lyapunov functions governing the same dynamics. To characterize their exact relation, consider a factored system of the form (50) with its ISS-Lyapunov function $V(n,\bar{n})$. The quasi-LTV representation of the system admits an ISS-Lyapunov function of the form

$$\begin{array}{c} \begin{array}{cc} V^{LTV}\left(n,\bar{n}\right) & =\sum_{i=1}^{m}\int_{{\bar{n}}_{i}}^{n_{i}}(\text{log}\;r-\text{log}\;{\bar{n}}_{i})dr\\ & =\sum_{i=1}^{m}(n_{i}\;\text{log}\;\frac{n_{i}}{{\bar{n}}_{i}}+{\bar{n}}_{i}-n_{i})=:\sum_{i=1}^{m}V_{i}^{LTV}\left(n_{i},{\bar{n}}_{i}\right) \end{array} \end{array}$$ (65)

so that we can write

$$\begin{array}{c} \begin{array}{cc} V(n,\bar{n}) & =\sum_{i=1}^{m}\int_{{\bar{n}}_{i}}^{n_{i}}(\text{log}\;({\hat{\theta }}_{i}\left(r\right)r)-\text{log}\;(\hat{\theta }\left({\bar{n}}_{i}\right){\bar{n}}_{i}))dr=\sum_{i=1}^{m}\int_{{\bar{n}}_{i}}^{n_{i}}(\text{log}\;{\hat{\theta }}_{i}\left(r\right)-\text{log}\;{\hat{\theta }}_{i}\left({\bar{n}}_{i}\right))dr\\ & +\sum_{i=1}^{m}\int_{{\bar{n}}_{i}}^{n_{i}}(\text{log}\;r-\text{log}\;{\bar{n}}_{i})dr=\sum_{i=1}^{m}\int_{{\bar{n}}_{i}}^{n_{i}}(\text{log}\;{\hat{\theta }}_{i}\left(r\right)-\text{log}\;{\hat{\theta }}_{i}\left({\bar{n}}_{i}\right))dr+V^{LTV}\left(n,\bar{n}\right). \end{array} \end{array}$$ (66)

**Remark 5.5.** *Since* $\sum_{i=1}^{m}{\bar{n}}_{i}=\sum_{i=1}^{m}n_{i}$ *we have that*

$$\begin{array}{c} V^{LTV}\left(n,\bar{n}\right)=\sum_{i=1}^{m}n_{i}\;\text{log}\;\frac{n_{i}}{{\bar{n}}_{i}}, \end{array}$$

*which is exactly the Kullback-Leibler divergence* $D_{KL}\left(n|\right|\bar{n})$ . *It is important to note that the Kullback-Leibler divergence is not a metric, since* $D_{KL}\left(n|\right|\bar{n})\neq D_{KL}(\bar{n}\left||n\right)$ *and it does not satisfy the triangle inequality. However, it is a nonnegative measure, meaning that it is nonnegative and zero if and only if* $n=\bar{n}$ *and it is often used to measure the “distance” of probability distributions for example in information theory and machine learning* [43].

While in general we are restricted to the above factorizations, in some special cases we may use a whole family of factorizations and corresponding Lyapunov functions. To illustrate this, consider an example when each $\theta_{i}\left(r\right)=\frac{r^{a_{i}}}{{\left(l+r\right)}^{b_{i}}}$ for some *l* > 0 and $a_{i}\in N$, $b_{i}\in N_{0}$, *a*_*i*_ ≥ *b*_*i*_ (these properties ensure that the functions *θ*_*i*_ are nondecreasing). Then, after the factorization described in Section 5.1, the Lyapunov function (45) becomes

$$\begin{array}{c} V^{\left(l,a,b\right)}\left(n,\bar{n}\right)=\sum_{i=1}^{m}(\left(a_{i}-b_{i}\right)\left({\bar{n}}_{i}-n_{i}\right)+a_{i}n_{i}\;\text{log}\;\frac{n_{i}}{{\bar{n}}_{i}}+b_{i}\left(l+n_{i}\right)\text{log}\;\frac{l+{\bar{n}}_{i}}{l+n_{i}}). \end{array}$$ (67)

We emphasize that (45) only depends on the *θ*_*i*_ functions, in this case parametrized with the *l*, *a*_*i*_, *b*_*i*_ values; that is, it is independent of the network structure and transition rate coefficients. We can also perform the factorization $\theta_{i}\left(r\right)={\tilde{\theta }}_{i}\left(r\right)\frac{r^{{\hat{a}}_{i}}}{{\left(l+r\right)}^{{\hat{b}}_{i}}}$ with ${\hat{a}}_{i}\in N$, ${\hat{a}}_{i}<a_{i}$, ${\hat{b}}_{i}\in N_{0}$, ${\hat{a}}_{i}\geq {\hat{b}}_{i}$ yielding the Lyapunov function $V^{\left(l,\hat{a},\hat{b}\right)}$ of the same form as in (67). This shows that the parameters *a* and *b* can be freely (apart from the constraints above) chosen in (67). We may also observe some interesting behaviour at the extrema of the parameters $\hat{b}$ and *l*, namely, that if we choose each ${\hat{b}}_{i}=0$ then the Lyapunov function in (67) is independent of *l*; that is, we have that

$$\begin{array}{c} V^{\left(l,\hat{a},0\right)}\left(n,\bar{n}\right)=\sum_{i=1}^{m}{\hat{a}}_{i}V_{i}^{LTV}\left(n_{i},{\bar{n}}_{i}\right). \end{array}$$ (68)

Moreover, letting *l*→∞ yields the convergence

$$\begin{array}{c} \underset{l\to \infty }{\text{log}\;}V^{\left(l,\hat{a},\hat{b}\right)}\left(n,\bar{n}\right)=\sum_{i=1}^{m}{\hat{a}}_{i}V_{i}^{LTV}\left(n_{i},{\bar{n}}_{i}\right) \end{array}$$ (69)

where $V_{i}^{LTV}$ is defined in (65).

#### Example 1.5: Family of Lyapunov functions of a generalized RFMR

Let us again consider a generalized version of the RFMR in the reduced state space from Fig 1. For a given initial condition *n*_0_ we can substitute *n*_3_ = *H*(*n*_0_) − *n*_1_ − *n*_2_, and thus the Lyapunov function restricted to the manifold $\{H\left(n\right)=H\left(n_{0})\right\}$ can be seen as a two dimensional function with local coordinates *n*_1_ and *n*_2_.

We set the capacities as *c*_1_ = *c*_2_ = *c*_3_ = 100 and *k*_12_ = 100, *k*_23_ = 60, *k*_31_ = 20. The system has transition rates as described above with each *a*_*i*_ = *b*_*i*_ = 3; that is, we have that

$$\begin{array}{c} \begin{array}{cc} K_{12}\left(n_{1},c_{2}-n_{2}\right) & =100\cdot \frac{n_{1}^{3}}{{\left(l+n_{1}\right)}^{3}}\cdot \frac{{\left(c_{2}-n_{2}\right)}^{3}}{{\left(l+c_{2}-n_{2}\right)}^{3}}\\ K_{23}\left(n_{2},c_{3}-n_{3}\right) & =60\cdot \frac{n_{2}^{3}}{{\left(l+n_{2}\right)}^{3}}\cdot \frac{{\left(c_{3}-n_{3}\right)}^{3}}{{\left(l+c_{3}-n_{3}\right)}^{3}}\\ K_{31}\left(n_{3},c_{1}-n_{1}\right) & =20\cdot \frac{n_{3}^{3}}{{\left(l+n_{3}\right)}^{3}}\cdot \frac{{\left(c_{1}-n_{1}\right)}^{3}}{{\left(l+c_{1}-n_{1}\right)}^{3}}. \end{array} \end{array}$$ (70)

**Fig 7:**
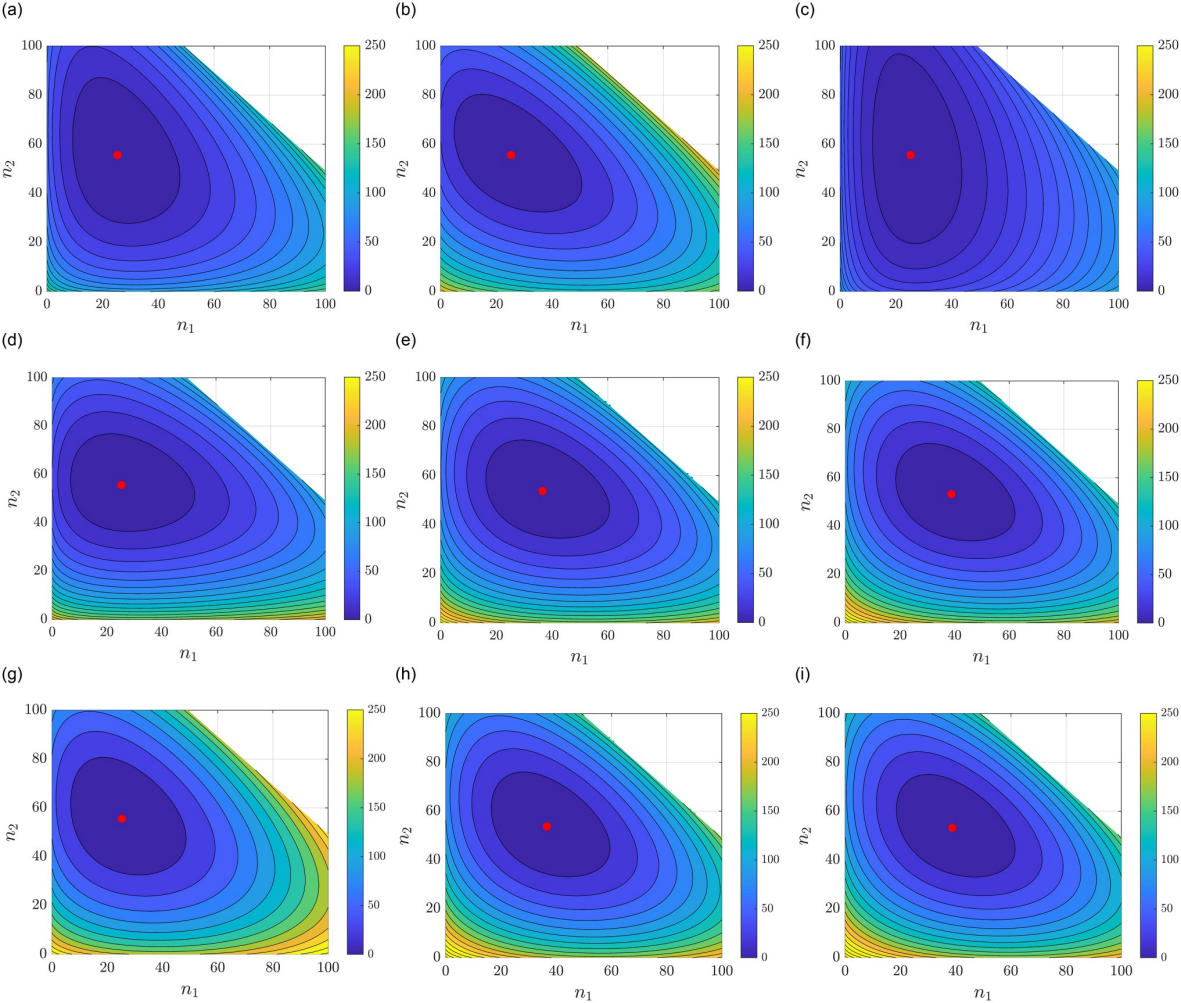
Comparison of Lyapunov functions for a generalized RFMR. (**a**) *l* = 25, $\hat{a}=[3\;3\;3],\hat{b}=[3\;3\;3]$. (**b**) *l* = 25, $\hat{a}=[1\;2\;3],\hat{b}=[0\;0\;1]$. (**c**) *l* = 25, $\hat{a}=[3\;1\;1],\hat{b}=[3\;1\;1]$. (**d**) *l* = 25, $\hat{a}=[2\;3\;2],\hat{b}=[2\;0\;2]$. (**e**) *l* = 100, $\hat{a}=[2\;3\;3],\hat{b}=[2\;0\;2]$. (**f**) *l* = 200, $\hat{a}=[2\;3\;2],\hat{b}=[2\;0\;2]$. (**g**) *l* = 25, $\hat{a}=[2\;3\;2],\hat{b}=[2\;0\;2]$. (**h**) *l* = 100, $\hat{a}=[2\;3\;2],\hat{b}=[2\;0\;2]$. (**i**) *l* = 200, $\hat{a}=[2\;3\;2],\hat{b}=[2\;0\;2]$.

The simulations were performed with *H*(*n*_0_) = 150. Fig 7a–7c show the Lyapunov function $V^{\left(l,\hat{a},\hat{b}\right)}$ for various choices of $\hat{a}$ and $\hat{b}$ with *l* = 25 fixed. The second and third rows demonstrate the convergence characterized in (69); Fig 7d–7f show $V^{\left(l,\hat{a},\hat{b}\right)}$ for increasing *l* values Fig 7g–7i shows $\sum_{i=1}^{m}{\hat{a}}_{i}V_{i}^{LTV}$ for the same increasing *l* values.

#### Example 3: Family of Lyapunov functions for a larger network

Let us consider a compartmental system with *m* = 100 compartments in the reduced state space. We assume that the transition rate functions are corresponding to Hill kinetics (modified intentionally to have different powers in the numerator and the denominator) and are of the form

$$\begin{array}{c} K_{ij}\left(n_{i},c_{j}-n_{j}\right)=k_{ij}\frac{n_{i}^{3}{\left(c_{j}-n_{j}\right)}^{3}}{(l+n_{i}^{2})(l+{\left(c_{j}-n_{j}\right)}^{2})} \end{array}$$ (71)

with *l* = 350. We assume that the only nonzero coefficients are

$$\begin{array}{c} \begin{array}{cc} & k_{i(i+1)}=20\;k_{i(i+2)}=18\;k_{i(i+3)}=16\;k_{i(i+4)}=14\\ & k_{i(i+5)}=12\;k_{i(i+6)}=10\;k_{i(i+7)}=8\;k_{i(i+8)}=6 \end{array} \end{array}$$ (72)

for *i* = 1, 2, …, *m*, where indices are understood as modulo *m*. Clearly this compartmental graph is strongly connected. Finally, we set capacities

$$\begin{array}{c} c_{1}=c_{2}=\cdots =c_{50}=50\;c_{51}=c_{52}=\cdots =c_{100}=100 \end{array}$$ (73)

Then the Lyapunov function (45) takes the form

$$\begin{array}{c} \begin{array}{cc} V_{Hill}^{\left(l,3,2\right)}\left(n,\bar{n}\right) & =\sum_{i=1}^{m}(\left({\bar{n}}_{i}-n_{i}\right)+3n_{i}\;\text{log}\;\frac{n_{i}}{{\bar{n}}_{i}}+n_{i}\;\text{log}\;\frac{{\bar{n}}_{i}^{2}+l}{n_{i}^{2}+l}\\ & +2\sqrt{l}(\text{atan}\frac{{\bar{n}}_{i}}{\sqrt{l}}-\text{atan}\frac{n_{i}}{\sqrt{l}})). \end{array} \end{array}$$ (74)

We can also factorize as $\theta_{i}\left(r\right)={\hat{\theta }}_{i}\left(r\right)\frac{r^{2}}{l+r^{2}}$, when (45) becomes

$$\begin{array}{c} \begin{array}{c} V_{Hill}^{\left(l,2,2\right)}\left(n,\bar{n}\right)=\sum_{i=1}^{m}(2n_{i}\;\text{log}\;\frac{n_{i}}{{\bar{n}}_{i}}+n_{i}\;\text{log}\;\frac{{\bar{n}}_{i}^{2}+l}{n_{i}^{2}+l}+2\sqrt{l}(\text{atan}\frac{{\bar{n}}_{i}}{\sqrt{l}}-\text{atan}\frac{n_{i}}{\sqrt{l}})). \end{array} \end{array}$$ (75)

**Fig 8:**
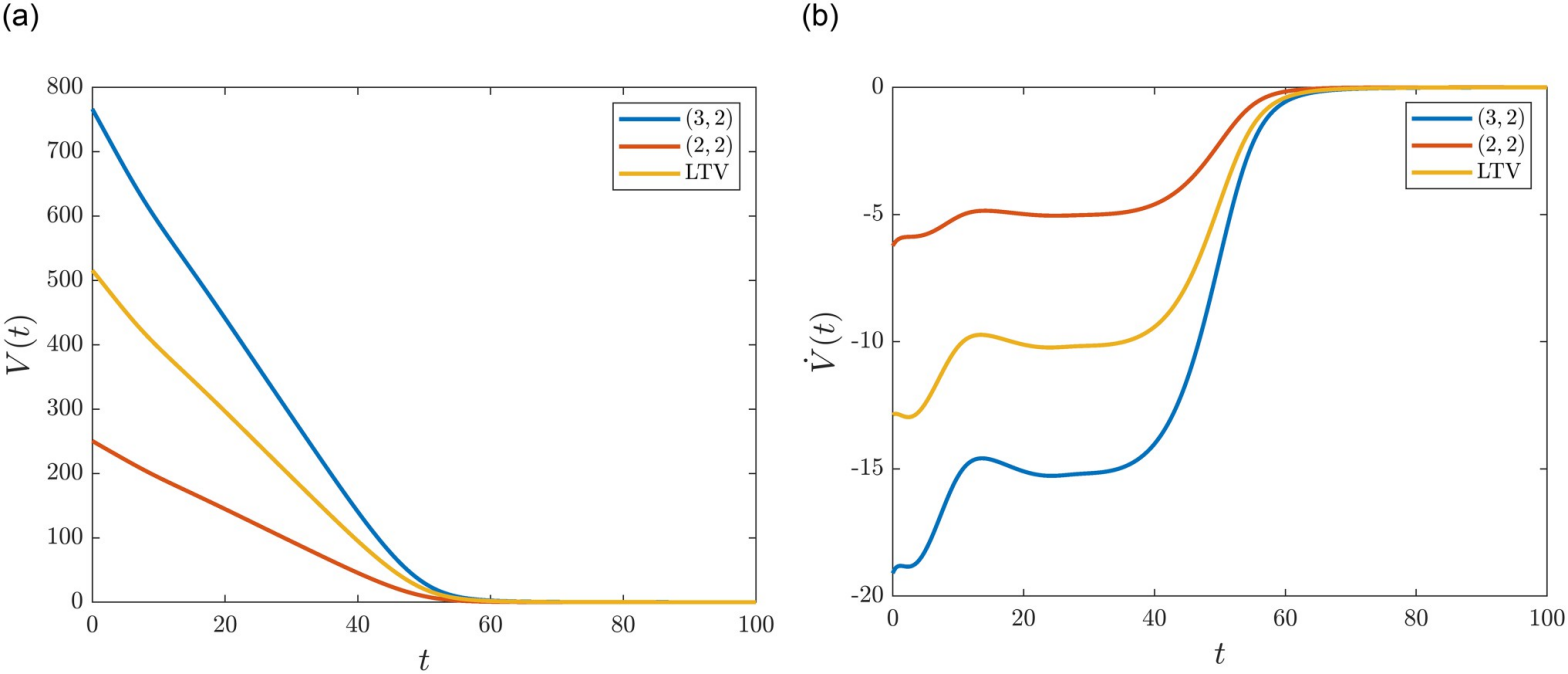
Time evolution and time derivative of Lyapunov functions obtained from various factorizations of the transition rates. **(a)** Time evolution of Lyapunov functions. **(b)** Derivative of Lyapunov functions.

Fig 8 shows the time evolution of Lyapunov functions $V_{Hill}^{\left(l,3,2\right)}$, $V_{Hill}^{\left(l,2,2\right)}$ and *V*^*LTV*^ and their time derivatives.

**Remark 5.6.** *In the above examples we restricted the factorizations to integer exponents so that we have real analytic transformations. However, the underlying dynamics is not changed through the factorizations and real analyticity is not directly used in the investigation of the ISS-Lyapunov function* (45). *Thus, as long as the factored* ${\hat{k}}_{ij}\left(t\right)$ *is piecewise locally Lipschitz (which holds after an arbitrarily short time in virtue of Remark 4.4), we can generalize* (67) *for other values as well; to be precise, we can use any* $0<{\hat{a}}_{i}\leq a_{i}$ and $0\leq {\hat{b}}_{i}\leq {\hat{a}}_{i}$ real numbers.

*Next, focusing on the Hill kinetics in* (71), *we note that while the denominator of the transformation* $\theta_{i}\left(r\right)=\frac{r^{3}}{l+r^{2}}$ in (71) *cannot be factorized we can rearrange the transformation as*

$$\begin{array}{c} \theta_{i}\left(r\right)=\frac{r^{3}}{l+r^{2}}=\underset{{\hat{\theta }}_{i}\left(r\right)}{\underset{︸}{\frac{r^{3-a_{i}}\left(l+r^{b_{i}}\right)}{l+r^{2}}}}\frac{r^{a_{i}}}{l+r^{b_{i}}}={\hat{\theta }}_{i}\left(r\right)\frac{r^{a_{i}}}{l+r^{b_{i}}} \end{array}$$ (76)

*where choosing* 0 < *a*_*i*_ ≤ 3 *and* 0 ≤ *b*_*i*_ ≤ *a*_*i*_ *ensures that the time-varying coefficient functions are piecewise locally Lipschitz. In this case the exact value of the integral in* (45) *involves the generalized hypergeometric function and generally cannot be expressed in a closed form. However, in some special cases (such as b*_*i*_ = 2 *above) we can calculate the integral explicitly; for example setting a*_*i*_ = 1.5 and *b*_*i*_ = 0.5 *yields*

$$\begin{array}{c} V_{Hill}^{\left(l,1.5,0.5\right)}\left(n,\bar{n}\right)=\sum_{i=1}^{m}(\left({\bar{n}}_{i}-n_{i}\right)+\frac{3}{2}n_{i}\;\text{log}\;\frac{n_{i}}{{\bar{n}}_{i}}+(n_{i}-l^{2})\text{log}\;\frac{\sqrt{{\bar{n}}_{i}}+l}{\sqrt{n_{i}}+l}+l(\sqrt{{\bar{n}}_{i}}-\sqrt{n_{i}})). \end{array}$$ (77)

#### Example 4: Competition for ribosomes in the cell

In this example we introduce a set of generalized ribosome flows connected by a finite pool of ribosomes to model competition in the cell. We follow [14], where the authors introduced a model for simultaneous translation and [16], where the authors generalized the model to include premature drop-off and attachment effects modeled with Langmuir kinetics. We will focus on the latter case and show that with a slight modification it can be formalized as a generalized ribosome flow model with a clear and natural compartmental interpretation. This demonstrates the usefulness and modeling power of generalized ribosome flows as one can prove various properties of many existing models of different conceptual levels. Moreover, our results show that many qualitative properties of the system carry over to more general settings, e.g. when the translation, drop-off and attachment rates are modeled with more sophisticated functions or when some (or all) rates are time-dependent.

For the sake of simplicity we will present this example in the reduced state space. Let us consider *N* mRNAs consisting of *m*_1_, *m*_2_, …, *m*_*N*_ number of sites. Let $n_{i}^{j}$ denote the continuous amount of ribosomes in the *i*th site of the *j* mRNA stand and let $c_{i}^{j}$ denote its capacity. Let *c*_*z*_ denote the capacity of the pool and *n*_*z*_ denote the amount of ribosomes in the pool. For the sake of notational simplicity let $n_{0}^{j}$ and $n_{m_{j}+1}^{j}$ also denote *n*_*z*_ and similarly for the capacities. Let the translation rate functions from the *i*th site the to (*i* + 1)th site on the *j*th mRNA be denoted as $K_{i(i+1)}^{j}$. Finally, let the detachment and attachment rates at the *i*th site of the *j*th mRNA be denoted respectively as $K_{iz}^{j}$ and $K_{zi}^{j}$. The attachment rate to the first site and the detachment rate from the last site will be called initiation rate and production rate, respectively. Then the dynamics of the model is given by:

$$\begin{array}{c} \begin{array}{cc} {\dot{n}}_{i}^{j} & =K_{(i-1)i}^{j}(n_{i-1}^{j},c_{i}^{j}-n_{i}^{j},t)-K_{i(i+1)}^{j}(n_{i}^{j},c_{i+1}^{j}-n_{i+1}^{j},t)\\ & +K_{zi}^{j}(n_{z},c_{i}^{j}-n_{i}^{j},t)-K_{iz}^{j}(n_{i}^{j},c_{z}-n_{z},t),\\ {\dot{n}}_{z} & =\sum_{j=1}^{N}(K_{m_{j}z}^{j}\left(n_{m_{j}}^{j},c_{z}-n_{z},t\right)-K_{z1}^{j}\left(n_{z},c_{1}^{j}-n_{1}^{j},t\right))\\ & +\sum_{j=1}^{N}\sum_{i=1}^{m_{j}}(K_{iz}^{j}\left(n_{i}^{j},c_{z}-n_{z},t\right)-K_{zi}^{j}\left(n_{z},c_{i}^{j}-n_{i}^{j},t\right)). \end{array} \end{array}$$ (78)

Thus, indeed, simultaneous translation with a finite pool can be described by a generalized ribosome flow. Clearly the following function defines a linear first integral

$$\begin{array}{c} H\left(n\right)=n_{z}+\sum_{j=1}^{N}\sum_{i=1}^{m_{j}}n_{i}^{j} \end{array}$$

and is a crucial factor in the dynamical analysis of the system.

**Remark 5.7.** *In* [16] *the authors consider the following special case*:

- *the capacity of each site is one; that is, each* $c_{i}^{j}=1$ ,
- *the translation rate are time-invariant and obey the mass-action law; that is, each* $K_{i(i+1)}^{j}\left(n_{i}^{j},1-n_{i+1}^{j},t\right)=\lambda_{i}^{j}n_{i}^{j}\left(1-n_{i+1}^{j}\right)$ *for some* $\lambda_{i}^{j}>0$ ,
- *the initiation and attachment rates are time-invariant and are given by* $K_{zi}^{j}\left(n_{z},1-n_{i}^{j},t\right)=\beta_{i}^{j}G_{j}\left(z\right)\left(1-n_{i}^{j}\right)$ for some $\beta_{i}^{j}\geq 0$ *and G*_*j*_(*z*) *continuously differentiable strictly increasing function with G*_*j*_(0) = 0,
- *the drop-off rates are time-invariant and are given by* $K_{iz}^{j}\left(n_{i}^{j},c_{z}-n_{z},t\right)=\alpha_{i}^{j}n_{i}^{j}$ for some $\alpha_{i}^{j}\geq 0$.

*Since the drop-off rates are donor controlled the pool does not have a predefined capacity and the amount of ribosomes in the pool are only bounded by H*(*n*(0)). *Therefore, this special case does not fit in our compartmental framework (although, as most of our results are a consequence of the linear first integral combined with the cooperativity of the system they can be generalized to include donor controlled terms as well). It is assumed that the authors consider this case to capture the fact that the capacity of the pool might be several orders higher than the actual number of ribosomes, and thus the dependence on the available space in the pool may be negligible. However, some physical meaning is lost with this assumption and it might in fact lead to less precise simulations*.

**Fig 9:**
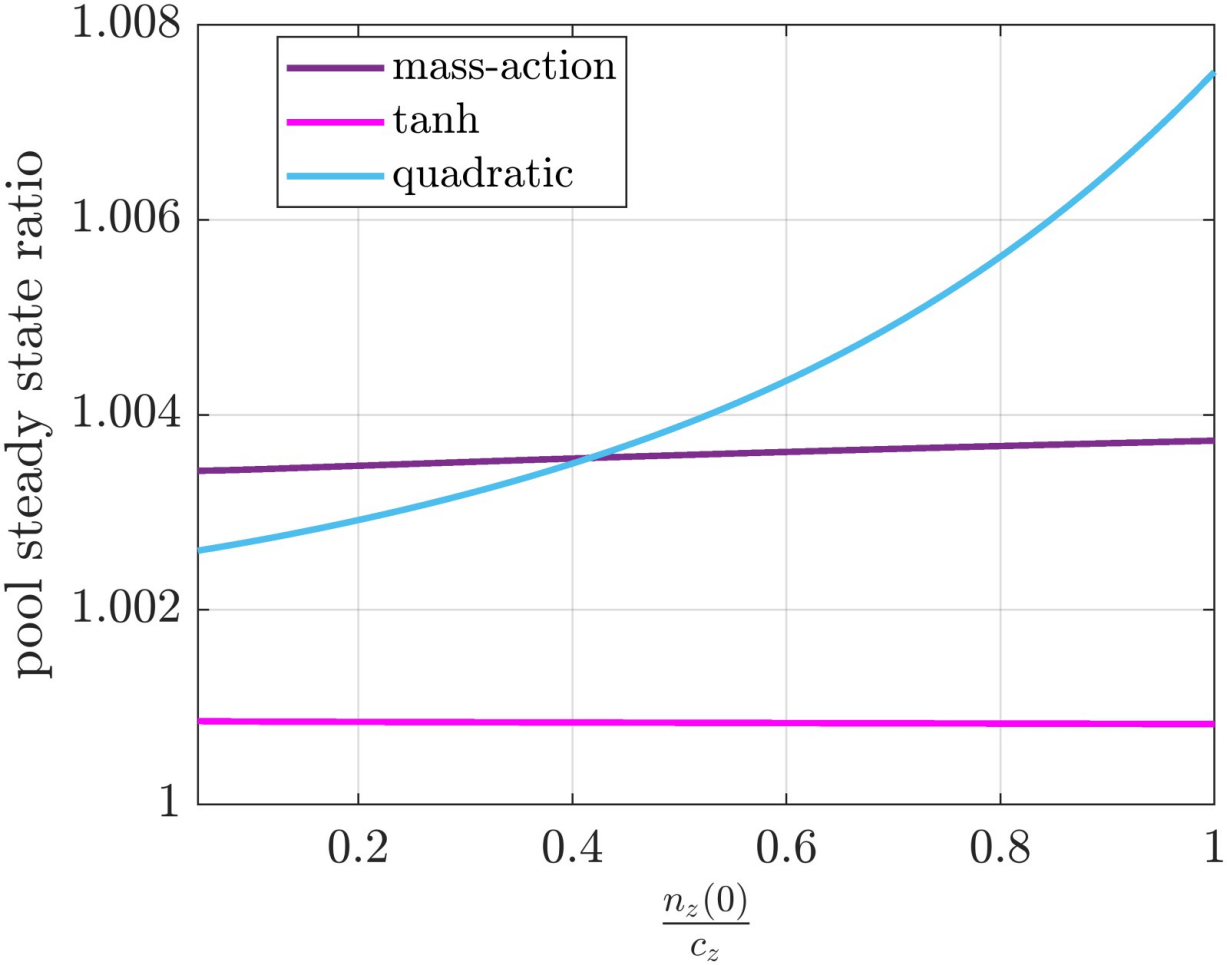
Steady state ratio of the donor controlled and the mass-action production rate for various initiation rates as a function of the ratio of the total number of ribosomes and the capacity of the pool.

*To see this, let us consider a network with N* = 10 *mRNAs with m* = 5 *sites. For the sake of simplicity let* $\lambda_{i}^{j}=\beta_{1}^{j}=\alpha_{5}^{j}=1$ *for each i and j, and assume that there are no premature drop-offs and attachments. We consider initation rates G*_*j*_(*z*) = *z*, *G*_*j*_(*z*) = tanh(*z*) *and G*_*j*_(*z*) = *z*^2^ *and set c*_*z*_ = 10^4^. *Since the equilibrium is unique on the level sets of the first integral we set each* $n_{i}^{j}=0$ and we only change *n*_*z*_(0). Fig 9 *shows the ratio of the steady state of the pool in the case of donor controlled and mass-action production rates as we increase the ratio* $\frac{n_{z}\left(0\right)}{c_{z}}$ *from* 5 ⋅ 10^−2^ *to* 1. *As expected, the steady state ratio is close to one for saturating rate functions and for n*_*z*_(0) ≪ *c*_*z*_. *However, the ratio can get higher when the total number of ribosomes have the same magnitude as the capacity; that is, the inaccuracy of the donor controlled kinetics increases. While this assumption might be valid for realistic parameters of ribosome flows in other TASEP based flow models (especially with non-saturating kinetics) it might be crucial to model these transitions accurately*.

**Fig 10:**
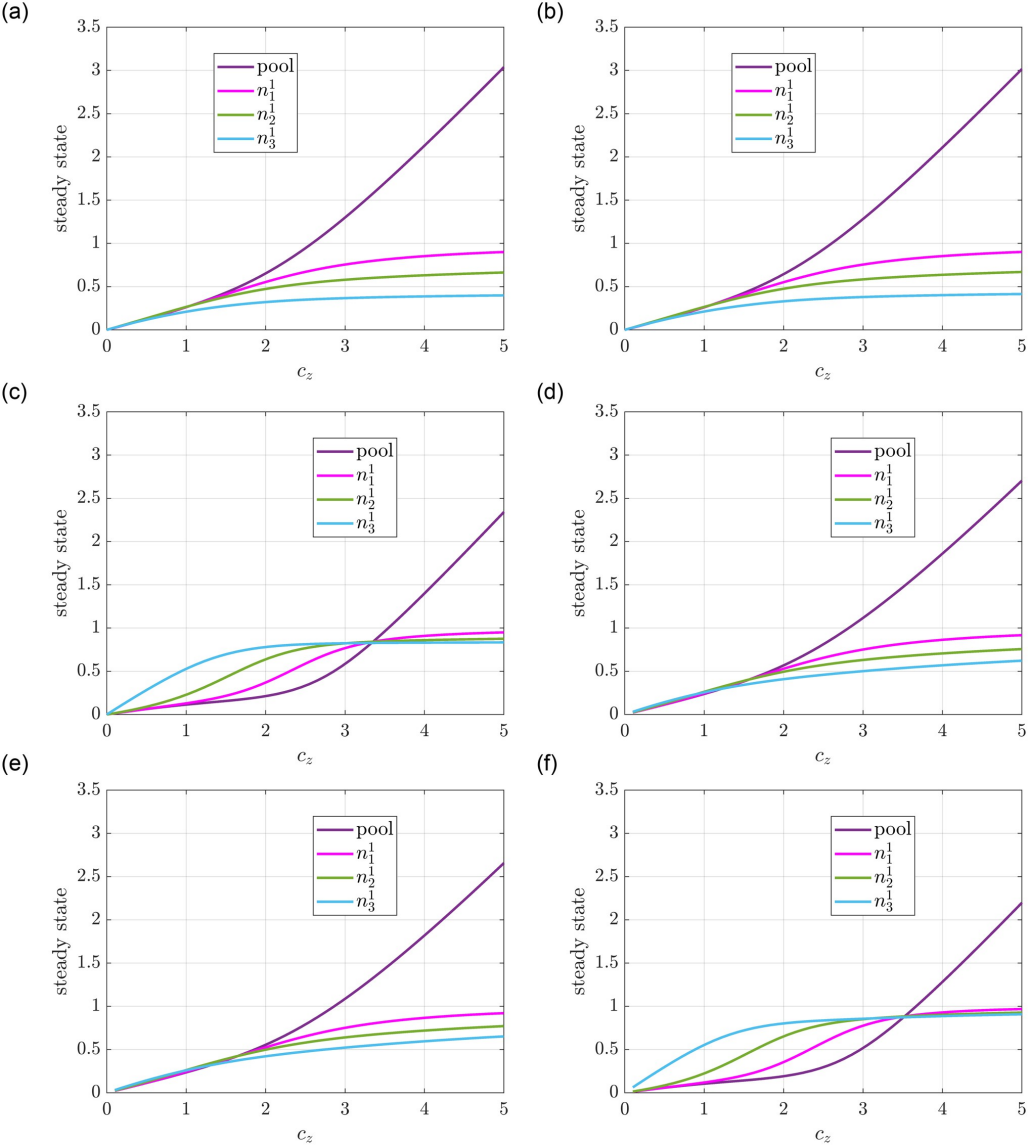
Steady state of a single mRNA strand in a pool modeled with an RFM and a GRFM with mass-action translation rate and drop-off rates, and attachment rate corresponding to different *G*_1_(*z*) functions. **(a)** RFM, *G*_1_(*z*) = *z*. **(b)** RFM, *G*_1_(*z*) = tanh(*z*). **(c)** RFM, $G_{1}(z)=\frac{\tanh(z)}{4+\tanh(z)}$. **(d)** GRFM, *G*_1_(*z*) = *z*. **(e)** GRFM, *G*_1_(*z*) = tanh(*z*). **(f)** GRFM, $G_{1}(z)=\frac{\tanh(z)}{4+\tanh(z)}$.

**Effect of the total number of ribosomes**. In the next simulation we follow [16] and we consider a single mRNA strand with *m*_1_ = 3 sites. The initiation rate is set to $\beta_{1}^{1}=1$ while the attachment rates are $\beta_{2}^{1}=0.1$ and $\beta_{3}^{1}=0$. The drop-off rates and production rate are set as $\alpha_{1}^{1}=0$, $\alpha_{2}^{1}=0.01$, $\alpha_{3}^{1}=1$. We assume that the translation rates obey the mass-action law with each $\lambda_{i}^{1}=1$. We set the initial values to $n_{j}^{1}=0$ and *n*_0_(*z*) = *c*_*z*_ as before. Fig 10 shows the steady state of the system as we increase *c*_*z*_ from 0 to 5 for various rate functions. One can see that in each case the mRNA saturates as we increase the number of ribosomes and the rest of the ribosomes are accumulated in the pool. Finally, the same effect as in Fig 9 can be observed; that is, the donor controlled detachment rates shift the steady state of the pool to higher values.

**Fig 11:**
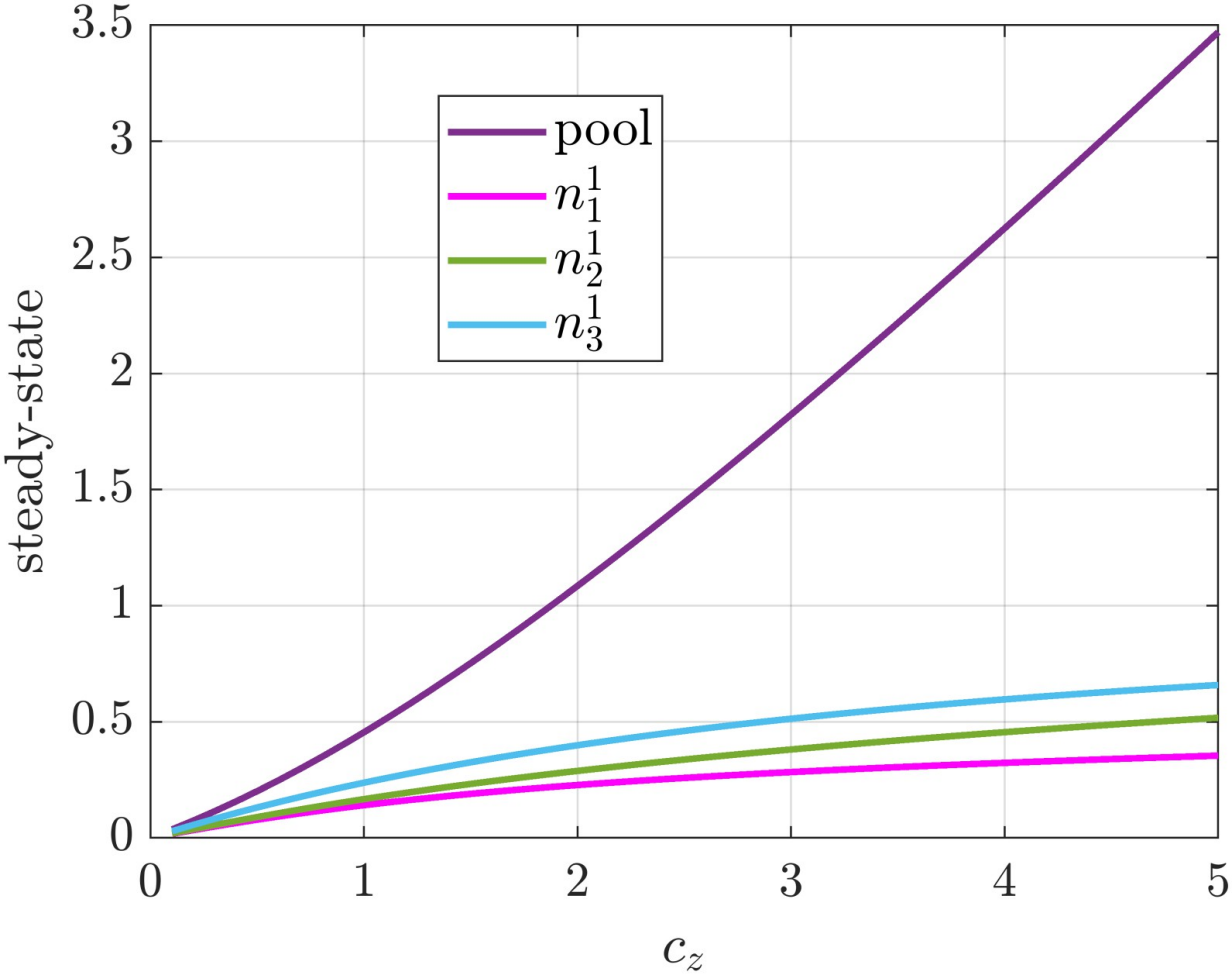
Steady state of a single mRNA strand in a pool modeled with a GRFM with mass-action translation rate, rational fraction drop-off rates, and saturating attachment rates.

We again emphasize the versatility of generalized ribosome flows as the initiation, translation, production, attachment and detachment rate function can be different on each site. For example let us consider a particular mRNA strand with saturating initation and attachment rates given by $K_{zi}^{1}\left(n_{z},n_{i}^{1}\right)=\beta_{i}^{1}\text{tanh}\left(n_{z}\right)\left(c_{i}^{1}-n_{i}^{1}\right)$, with mass-action translation rates and with production and drop-off rates given by $K_{iz}^{1}\left(n_{i}^{1},n_{z}\right)=\alpha_{i}^{1}\cdot \frac{n_{i}^{1}}{1+n_{i}^{1}}\cdot n_{z}^{3}$. Fig 11 shows evolution of the steady states as we increase *n*_*z*_(0) = *c*_*z*_ as before. As expected the steady states of the mRNA sites are moved to lower values.

## 6 Conclusions

The dynamical properties of generalized ribosome flow models models with arbitrary compartmental graph structure and general time-varying transition rates were studied in this paper. The analysis is based on the deterministic CRN representation of such systems which has a transparent physical meaning by tracking the amounts (concentrations) of available objects and free spaces, respectively, in each compartment. Our framework includes several important models from the literature including the RFMR [13], and its generalizations like the RFMRD [15]. As demonstrated in Example 4, our framework can describe complex phenomena like competition for ribosomes in a cell through a set of tubular flow models connected with a pool of finite capacity. The obtained model (with a slight modification due to physical considerations) includes previously published pool models such as [16]. It was shown that the deficiency of the obtained kinetic model form is equal to the number of chordless cycles in the undirected reaction graph of the system. Furthermore, it was proved that time-varying generalized ribosome flows are persistent under mild regularity assumptions on the transition rates, and a wide set of reaction rates satisfying this assumption was characterized, containing well-known examples such as mass-action type rates. It was shown that the studied models can be embedded in at least two ways into the class of rate-controlled biochemical networks originally described in [22]. This embedding allows us to prove stability with entropy-like logarithmic Lyapunov functions known from the theory of CRNs. It was illustrated that the non-unique factorization of the rate functions gives rise to a whole family of various possible Lyapunov functions. Finally, periodic model behaviour was also studied, where we showed that trajectories with the same overall initial mass and periodic transition rates having the same period (but possibly different phase) converge to a unique periodic solution. The generality of our setting allows the efficient extension of the proposed results to ribosome flows open to the environment, for example as in [44, 45]. It also allows the generalization of other qualitative results in the literature, for example translation rate maximization [15, 46]. We emphasize that the persistence and stability results, and even the basic logarithmic Lyapunov function candidate in Eq (45) are independent of the potentially uncertain time varying parameters (rate coefficients) of the model. Besides the theoretical aspects, these improvements may efficiently support structural design or control of compartmental models with bounded capacities, which is planned to be addressed in our future work.
